# NF-κB in biology and targeted therapy: new insights and translational implications

**DOI:** 10.1038/s41392-024-01757-9

**Published:** 2024-03-04

**Authors:** Qing Guo, Yizi Jin, Xinyu Chen, Xiaomin Ye, Xin Shen, Mingxi Lin, Cheng Zeng, Teng Zhou, Jian Zhang

**Affiliations:** 1 Department of Medical Oncology, Fudan University Shanghai Cancer Center, No. 270, Dong’an Road, Shanghai, 200032 China; 2 Department of Oncology, Shanghai Medical College, Fudan University, Shanghai, China; 3 State Key Laboratory of Oncogenes and Related Genes, Renji-Med-X Stem Cell Research Center, Shanghai Cancer Institute & Department of Urology, Ren Ji Hospital, School of Medicine and School of Biomedical Engineering, Shanghai Jiao Tong University, Shanghai, 200127 PR China; 4 Department of Cardiology, the First Affiliated Hospital of Sun Yat-Sen University, 58 Zhongshan 2nd Road, Guangzhou, 510080 China; 5 Department of Neurology, Ruijin Hospital, Shanghai Jiao Tong University School of Medicine, Shanghai, China

**Keywords:** Oncology, Cancer

## Abstract

NF-κB signaling has been discovered for nearly 40 years. Initially, NF-κB signaling was identified as a pivotal pathway in mediating inflammatory responses. However, with extensive and in-depth investigations, researchers have discovered that its role can be expanded to a variety of signaling mechanisms, biological processes, human diseases, and treatment options. In this review, we first scrutinize the research process of NF-κB signaling, and summarize the composition, activation, and regulatory mechanism of NF-κB signaling. We investigate the interaction of NF-κB signaling with other important pathways, including PI3K/AKT, MAPK, JAK-STAT, TGF-β, Wnt, Notch, Hedgehog, and TLR signaling. The physiological and pathological states of NF-κB signaling, as well as its intricate involvement in inflammation, immune regulation, and tumor microenvironment, are also explicated. Additionally, we illustrate how NF-κB signaling is involved in a variety of human diseases, including cancers, inflammatory and autoimmune diseases, cardiovascular diseases, metabolic diseases, neurological diseases, and COVID-19. Further, we discuss the therapeutic approaches targeting NF-κB signaling, including IKK inhibitors, monoclonal antibodies, proteasome inhibitors, nuclear translocation inhibitors, DNA binding inhibitors, TKIs, non-coding RNAs, immunotherapy, and CAR-T. Finally, we provide an outlook for research in the field of NF-κB signaling. We hope to present a stereoscopic, comprehensive NF-κB signaling that will inform future research and clinical practice.

## Introduction

In 1986, Ranjan Sen and David Baltimore first identified the nuclear factor in B lymphocytes binding to the kappa enhancer of the gene encoding the κ light- chain of immunoglobulin by electrophoretic migration assays of end-labeled DNA fragments and named it nuclear factor binding near the κ light- chain gene in B cells or NF-κB.^1,2^ Over the next 3 years, David Baltimore’s laboratory successively uncovered the significance of kappa enhancer’s protein-binding site κB in promoting transcriptional activity and inducibility in B cells, as well as NF-κB as a molecule involved in several pathways.^3–5^ The involvement of NF-κB in inflammation and immune responses is indisputable, and this is the most important role that NF-κB signaling plays in biology. The understanding of NF-κB signaling should not be limited to its “results”. It is more meaningful to investigate the intricate biological mechanisms by which NF-κB signaling induces alterations in molecules, cells, tissues, and even organisms across diverse species and diseases. Additionally, it is crucial to comprehend the dualistic nature of NF-κB signaling, which can act as both a “foe” and a “friend”. The greater the depth and breadth of our comprehension of NF-κB signaling, the more assured we become in our ability to exploit this pathway for gene manipulation, modulation of cellular behavior, and therapeutic intervention. We will commence by presenting the biological underpinnings of NF-κB signaling and elucidate the mechanisms of its self-regulation and crosstalk with other pathways. Further, we provide a comprehensive overview of the role of NF-κB signaling in the pathogenesis of diverse organ systems and discuss therapeutic strategies targeting this pathway, which will be beneficial to better understand the research process of NF-κB signaling.

## The history and development of NF-κB signaling

**Fig. 1:**
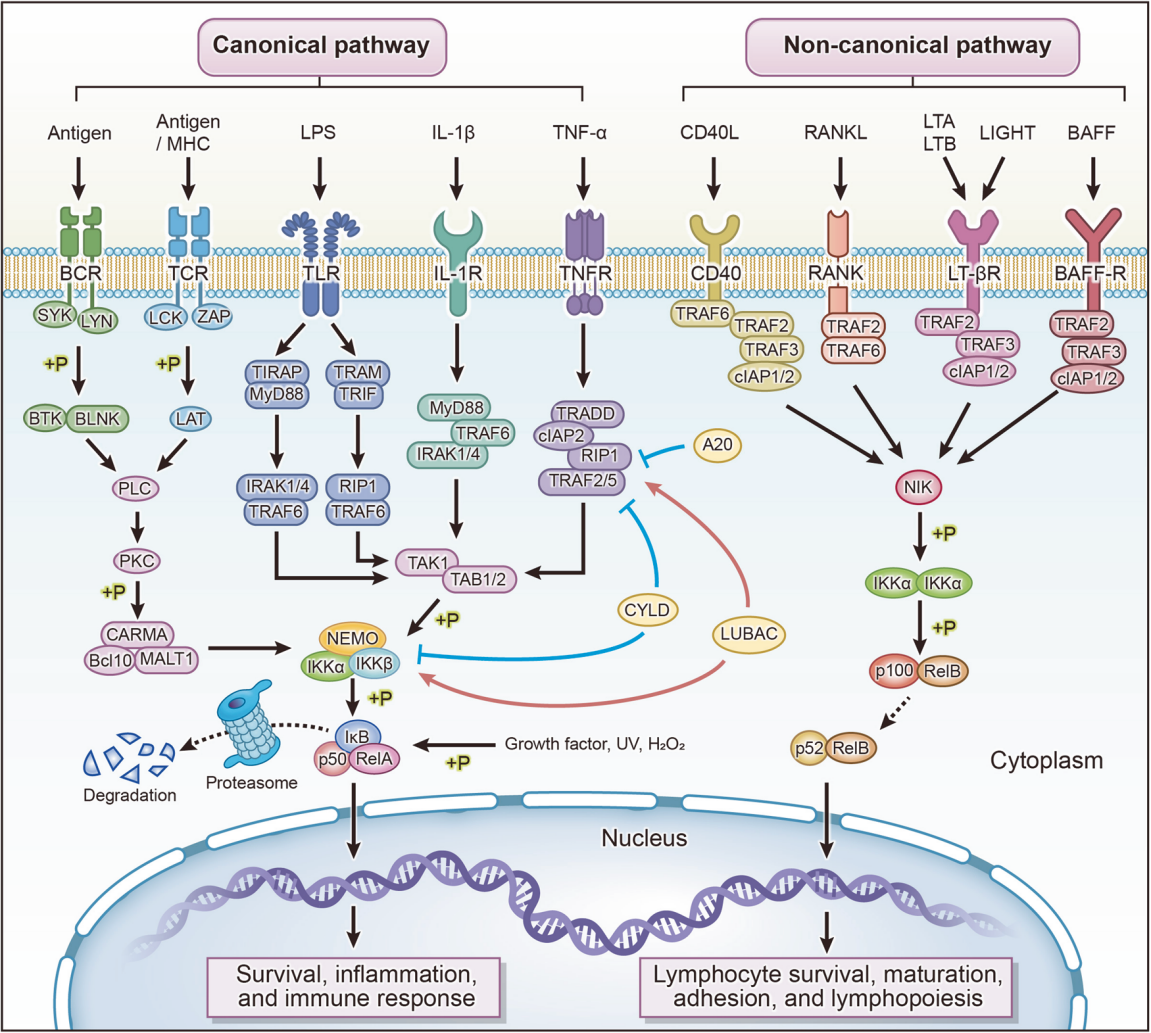
Overview of canonical and non-canonical NF-κB signaling. Canonical NF-κB signaling is primarily activated by BCR, TCR, TLR, IL-1R, and TNFR. BCR and TCR initiate a multistage enzymatic reaction that activates the CARMA1/BCL-10/MALT1 complex. TLR, IL-1R, and TNFR primarily promote activation of the TAK1/TAB complex. Activated CARMA1/BCL-10/MALT1 complex and TAK1/TAB complex phosphorylate the IKKα/IKKβ/NEMO (IKKγ) complex. IKKα and IKKβ phosphorylate IκBα, leading to its ubiquitination and subsequent proteasomal degradation. This results in the release of p50/RelA, which acts as a transcription factor to activate the transcription of target genes. Canonical NF-κB signaling primarily promotes cell survival and mediates inflammatory and immune responses. In non-canonical NF-κB signaling, CD40, RANK, LT-βR, and BAFF-R activate NIK, which further phosphorylates IKKα and promotes the degradation of p100 to p52. The p52 subunit then binds to RelB and undergoes nuclear translocation, promoting lymphocyte generation, survival, maturation, and adhesion. A20 TNF alpha-induced protein 3, BAFF B lymphocyte activating factor, BAFF-R B lymphocyte stimulating factor receptor, Bcl10 B cell leukemia/lymphoma 10, BCR B-cell receptor, BLNK B cell linker, BTK Bruton tyrosine kinase, CARMA1 caspase recruitment domain family member 11, CD40L CD40 ligand, CYLD cylindromatosis, IAP inhibitor-of-apoptosis protein, IKK I-kappaB kinase, IL-1 interleukin 1,IL-1R interleukin 1 receptor, IRAK interleukin 1 receptor-associated kinase, IκB IkappaB protein, LAT linker for activation of T cells, LCK lymphocyte cell-specific protein tyrosine kinase, LIGHT tumor necrosis factor ligand superfamily member 14, LPS lipopolysaccharide, LTA lymphotoxin alpha, LTB lymphotoxin beta, LT-βR lymphotoxin beta receptor, LUBAC linear ubiquitin chain assembly complexes, LYN LYN proto-oncogene, Src family tyrosine kinase, MALT1 MALT1 paracaspase, MHC major histocompatibility complex, MyD88 MYD88 innate immune signal transduction adapter, NEMO inhibitor of nuclear factor kappa-B kinase subunit gamma, NIK NF-κB-inducing kinase, PKC protein kinase C, PLC phospholipase C, RANK receptor activator of NF-KappaB, RANKL receptor activator of NF-KappaB ligand, RIP1 receptor-interacting serine/threonine-protein kinase 1, SYK spleen associated tyrosine kinase, TABTAK1-associated binding protein, TAK1 TGF-beta activated kinase 1, TCR T-cell receptor, TIRAP TIR domain containing adapter protein, TLR toll-like receptor, TNF tumor necrosis factor, TNFR TNF receptor, TRADD tumor necrosis factor receptor type 1-associated DEATH domain protein, TRAF tumor necrosis factor receptor-associated factor, TRAM TRIF-related adapter molecule, TRIF toll-like receptor adapter molecule 1, ZAP tyrosine-protein kinase ZAP-70

The mammalian NF-κB transcription factor family consists of five members, namely NF-κB1 (p105/p50), NF-κB2 (p100/p52), p65 (RELA), V-Rel reticuloendotheliosis viral oncogene homolog B (RELB), and c-REL. Due to the sharing of the conserved Rel homology domain (RHD), any two members of the NF-κB transcription factor family can form homo- or heterodimers, which bind to IκB and sequester in the cytoplasm in an inactive form, with p65/p50 being the most common dimerization form.^6^ Specific functions of several NF-κB complex types are involved in the development of regulatory T cells.^7^ RELA, RELB, and c-REL harbor transcriptional activation structural domains (TAD) with transcriptional activation activity. While p50 and p52 do not contain TAD and their homodimers are transcriptional repressors, p50 and p52 form heterodimers with TAD-containing family members to further stimulate transcription or alter the specificity of the κB site.^8,9^ (Fig. 1)

The I-kappaB kinase (IKK) kinase complex constitutes a key component of the NF-κB signaling cascade.^10^ The IKK complex consists of IKKα, IKKβ, and NEMO (IKKγ), of which IKKα and IKKβ are the kinases and IKKγ is the subunit that exerts the regulatory function. IKKα and IKKβ share 50% sequence identity, and both molecules include an amino-terminal kinase domain, a helix-loop-helix (HLH) responsible for regulating IKK kinase activity, and a leucine zipper (LZ) mediating kinase dimerization.^10^

### Canonical NF-κB pathway

#### Components of canonical NF-κB pathway

##### NF-κB family

The precursor molecule p105 undergoes ubiquitination upon induction by the ubiquitin ligase Kip1 ubiquitination-promoting complex subunit 1 (KPC1), followed by proteasomal disassembly into the active form p50, and the nuclear localization sequence (NLS) masked by the remote structural domain of the p110 precursor is exposed, allowing cytoplasmic/nuclear signaling to proceed.^11,12^ When combined with RelA to form a heterodimer, p50 participates in the transmission of canonical NF-κB signaling.

##### IκB family

The IκB (IkappaB protein, inhibitor of NF-κB) family comprises p100, p105, IκBα, IκBβ, IκBε, IκBζ, BCL-3, and IκBNS.^13^ IκB binds to NF-κB through 3–8 ankyrin repeats at the C-terminus, masking the nuclear localization sequence (NLS) of NF-κB and inhibiting its activity. The N-terminus contains phosphorylation and ubiquitination sites, which are signal-responsive regions involved in the induced degradation of IκB.

IκBα, IκBβ, and IκBε are present as typical IκB proteins in the cytoplasm of resting cells, and stimulation may induce degradation and resynthesis of typical IκB proteins.^8^ Unlike the rapid and transient activation of IκBα-mediated NF-κB signaling, IκBβ sustainably activates NF-κB and maintains long-term expression of pro-inflammatory target genes such as tumor necrosis factor-α (TNF-α) through p65:c-Rel heterodimer.^14,15^ IκBγ is mainly found in lymphocytes.^16^ Synthesis of IκBα is specifically induced by the p65 subunit of NF-κB. IκBα binds to the p65 subunit and is present in the cytoplasm, inhibiting the transcription factor activity of NF-κB.^17–20^ In response to activated IKK, IκBα is phosphorylated at serine/threonine residues and degraded. The cytoplasmic complex composed of NF-κB and IκB dissociates, and NF-κB is released into the nucleus where it activates the transcription of downstream target genes.^17–20^

Cell activation stimulates the creation of atypical IκB proteins (IκBζ, BCL-3, and IκBNS), which then play their respective roles in the nucleus.^8,21–25^ Different from the above IκB family members, the proto-oncogene Bcl-3 can also bind tightly to p50/p52 homodimers and DNA in the nucleus to transactivate through the κB motif.^26–28^ Bcl-3 is not only an inhibitor that sequesters NF-κB to the cytoplasm and inhibits its activity, but also participates in the transcriptional process as a transcriptional coactivator.^8^ IκB acts on the transcription of NF-κB by affecting the production, stability, and reactivity of NF-κB complexes.^8^

##### IKK family

Amino acid regions (aa 705–743) at the carboxyl terminus of IKKα and IKKβ mediate interaction with NEMO. IKK performs a dual function of activating NF-κB and inhibiting the cell death pathway.^7^ In unstimulated cells, IκB inhibits the DNA-binding activity of NF-κB dimers and keeps them homeostatic localization in the cytoplasm. In stimulated cells, phosphorylation of serine residues located in IKKα proteins 176 and 180 and serine residues in IKKβ proteins 177 and 181 leads to changes in protein conformation and activation of the kinase.

Phosphorylation of IKKβ is required for canonical NF-κB signaling, and TGF-beta activated kinase 1 (TAK1) is responsible for IKKβ phosphorylation upon binding to the cofactor TAK1-associated binding protein (TAB1/2/3). IκB is phosphorylated by the active IKK complex, which causes ubiquitination and eventual destruction of IκB. NF-κB dimer is released and nuclear transposed, binding to the κB site in the promoter or enhancer and activating the transcription of specific genes. Therefore, IKK is a key regulatory event for NF-κB activation. In addition, IKKα and IKKβ are also involved in the phosphorylation of the p65 subunit.^29–32^

NEMO is also necessary for canonical NF-κB signaling activation. The IKK-binding domain (IBD) at the N-terminal of NEMO binds to the NEMO-binding domain (NBD) of IKK, and the C-terminal end mediates interactions with upstream signaling molecules, such as RIP, which promotes oligomerization of NEMO and phosphorylation of IKKα/β, and plays a crucial role in TNF-α, interleukin (IL)-1-activated NF-κB signaling.^33–37^ NEMO acts as a scaffold in the recruitment of IκBα by IKKβ.^38^ In the case of the NEMO mutation, IKKβ undergoes hyperphosphorylation upon activation by IL-1 but fails to recruit IκB.^38^ Yu et al. found that ubiquitin carboxy-terminal hydrolase 16 (USP16) competitively binds IKKα and IKKβ to NEMO, thereby inhibiting the interaction of IKKβ with NEMO.^39^ During antigen-induced activation of NF-κB signaling in T or B cells, IKK is phosphorylated in response to stimulus-dependent conformational changes or oligomerization activation, which may be related to NEMO.

#### Activation and regulation of canonical NF-κB pathway

NF-κB signaling may be activated by a diverse range of stimuli, including bacterial and viral products, cytokines, ultraviolet and ionizing radiation, growth factors, reactive oxygen species, and oncogenic stresses.^6,40^ Immune cells utilize unique, dynamic quantitative signal signatures that stimulate NF-κB signaling outside the cell or intracellular to transmit important biological information about the microenvironment.^41^ Dangerous stimuli such as pathogen invasion initiate innate immune responses, and dynamically encode specific information such as ligand dose, duration, and distance through wave propagation of NF-κB signaling, forming gene expression regions in response cells.^41,42^ The main activators of canonical NF-κB signaling include TNF-α, interleukin (IL)-1β, lipopolysaccharide (LPS), and antigen. These activators will bind to cell surface receptors and trigger the activation of NF-κB signaling in response to multiple bridging proteins. In the following section, we will describe the conduction and regulation process of canonical NF-κB signaling induced by different stimuli respectively.

##### TNF-α induced canonical NF-κB pathway

Hailing Hsu et al. discovered the tumor necrosis factor receptor type 1-associated DEATH domain (TRADD), which interacts with the intracellular structural domain of the TNF receptor 1 (TNFR1), in 1995, and suggested that TRADD is implicated in TNF-induced NF-κB signaling.^43^ Subsequently, the team found that TRADD directly interacts with the ubiquitin ligase tumor necrosis factor receptor-associated factor (TRAF2) and protein kinase receptor-interacting serine/threonine-protein kinase (RIP), activating NF-κB signaling.^44,45^ Further investigations have revealed that TRAF2 exhibits a higher binding affinity towards TRADD for signaling, rather than TNFR1, and impedes apoptosis by recruiting inhibitor-of-apoptosis proteins (clAPs).^46^ Lipid rafts, which are membrane microdomains enriched in cholesterol and sphingolipids, serve as a structural foundation for the assembly of TNFR1-RIP-TRADD-TRAF2 complexes.^47^ Within these complexes, where TNFR1 and RIP are ubiquitinated for NF-κB signaling.^47^ The TNFR1-RIP-TRADD-TRAF2 complex plays a crucial role in regulating cell survival and apoptosis^48^^,49^, and when TNFR1-mediated signaling successfully activates the complex and NF-κB signaling, the cells will survive in the presence of FLICE inhibitory proteins (FLIP, Caspase-8 inhibitor), and conversely lead to cell death.^14,15^

TNF alpha-induced protein 3 (A20) and CYLD lysine 63 deubiquitinase (CYLD) are key deubiquitinases in the downregulation of NF-κB signaling.^50^ A20 is an NF-κB signaling inhibitor comprising two structurally independent domains. The N-terminus of A20 functions as an ovarian tumor family deubiquitinating enzyme with linear linkage specificity (OTULIN), which specifically cleaves K63-linked ubiquitin chains from RIP. The C-terminus of A20 acts as a ubiquitin ligase for K48-linked polyubiquitination, leading to the proteasomal degradation of RIP.^51^ CYLD clears non-K48-linked polyubiquitin chains on a range of NF-κB signaling proteins and negatively regulates TRAF2- or TRAF6-mediated IKK activation through deubiquitination.^50,52,53^ The E3 ligase linear ubiquitin chain assembly complexes (LUBAC) consist of a catalytic HOIL-interacting protein (HOIP) and a regulated Shank-associated RH domain-interacting protein (SHARPIN) and Heme-oxidized IRP2 ubiquitin ligase 1 (HOIL-1L) composition.^54–56^ Since LUBAC promotes linear ubiquitination of NEMO and RIP1, it is considered a key mechanism for the activation in response to specific stimuli or overactivation of NF-κB signaling.^57,58^

##### IL-1β induced canonical NF-κB pathway

The investigation into interleukin 1 receptor-associated kinase (IRAK) as an essential component for IL-1 activation of NF-κB signaling originated from the discovery by Zhaodan Cao et al. that IRAK promptly binds to and phosphorylates the interleukin 1 receptor, type I (IL-1RI) in tool cells (HEK 293 and HeLa).^59,60^ IRAK shares similarity in the primary amino acid sequence with Pelle, a protein kinase essential for activation of the Drosophila NF-κB homolog.^59,60^ In the same year, the team found that TRAF6, a member of the TRAF family, is induced by IL-1 to bind with IRAK and is rapidly recruited to IL-1R, implying that TRAF6 is also involved in IL-1-NF-κB signaling.^61^ Marta Muzio’s team identified IRAK-2 and MYD88 innate immune signal transduction adapter (MyD88) as mediators for IL-1R-induced NF-κB signaling, and MyD88 serves as a signal transduction adapter to mediate the binding of IRAK to IL-1R, which provides possible targets for the treatment of inflammatory diseases.^62–64^ MyD88 recruits IRAK1 and IRAK4 via death structural domain, and IRAK4 triggers autophosphorylation and subsequent dissociation of IRAK1. TRAF6 functions as a signal transducer primarily involved in canonical NF-κB signaling activated by IL-1 and toll-like receptor (TLR). The activation of IKK by TRAF6 is achieved through the synthesis of lysine-63 (K63)-linked polyubiquitin chains catalyzed by the ubiquitin ligases Ubc13 and Uev1A, and the TAK1/TAB1/TAB2 protein kinase complex phosphorylates and activates IKK with the assistance of the polyubiquitin chains.^65–67^

##### LPS induced canonical NF-κB pathway

TLR recognizes molecules such as LPS, DNA, and RNA from viruses, bacteria, and fungi as sensors for detecting possible infections and initiating an immune cascade response for host defense.^68^ The toll-IL-1 receptor (TIR) structural domain of TLR4 recruits the TLR adapter molecule MyD88 and the TIR domain containing adapter protein (TIRAP), which subsequently activate IRAK1/4 and TRAF6, and involved in TLR-stimulated NF-κB signaling are also toll-like receptor adapter molecule 1 (TRIF) and TRIF-related adapter molecule (TRAM), both signaling modes are dependent on interaction with TLR4.^69,70^ The ubiquitin-conjugating enzyme complex composed of TRAF6/ Ubc13/ Uev1A catalyzes the formation of K63-linked polyubiquitin chain, which activates TAK1.^65,71^

##### Antigen induced canonical NF-κB pathway

T cells and B cells are the main cell types responsible for adaptive immune. T-cell receptor (TCR) is activated upon binding to the major histocompatibility complex (MHC)-antigen peptide complex. TCR is first recruited through the intracellular structural domains of CD4 and CD8 by lymphocyte cell-specific protein tyrosine kinase (LCK) to phosphorylate immunoreceptor tyrosine activation motifs (ITAM) and activate the tyrosine-protein kinase ZAP-70.^72,73^ ZAP-70 phosphorylates the activating linker for the activation of T cells (LAT), which promotes the recruitment of multiple junction proteins and effector molecules including phospholipase C γ1 (PLCγ1) and the formation of the LAT signalosome complex.^74^ PLCγ1 catalyzes the synthesis of diester glycerol and inositol (1,4,5)-trisphosphate as second messengers that trigger mitogen-activated protein kinase, protein kinase Cθ (PKCθ) and calmodulin phosphatase.^73,75^ The B-cell receptor (BCR) upon binding to antigen, first recruits spleen associated tyrosine kinase (SYK) and SRC proto-oncogene, non-receptor tyrosine kinase (SRC), which determines the initiation of BCR signaling and subsequent conductance efficiency.^76^ As a member of the SRC kinase family, LYN proto-oncogene, Src family tyrosine kinase (LYN) phosphorylates tyrosine residues of ITAM and SYK in CD79A and CD79B.^76,77^ B cell linker (BLNK), a substrate for SYK, promotes the recruitment of Bruton tyrosine kinase (BTK) and PLCγ2, and BTK phosphorylation activates PLCγ2 and PKCβ, which leads to intracellular calcium mobilization and activation of NF-κB signaling.^76^ Activated PKCθ and PKCβ recruit caspase recruitment domain family member 11 (CARMA1), B cell leukemia/lymphoma 10 (Bcl-10), and MALT1 paracaspase (MALT1), and the complex composed of CARMA1/BCL-10/MALT1 was found to be active in NF-κB and c -Jun N-terminal kinase (JNK) signaling, which mediates immune cell activation, proliferation, and differentiation, and its aberrant expression has been associated with autoimmune diseases and lymphoma formation.^78,79^ PKCθ and PKCβ mediate the interaction between TAK1 and CARMA1 and recruit IKK, which activates downstream NF-κB signaling.^76,80^

Termination of NF-κB is associated with nuclear degradation and re-localization of NF-κB subunits, and dissociation of coactivators.^8^ NF-κB signaling promotes the expression of IκBα, which is newly synthesized to enclose it in the cytoplasm by conjugation with the NF-κB dimer, thereby promoting the termination of transcriptional responses, and plays a significant role in the negative feedback loop of NF-κB signaling.^81^

### Non-canonical NF-κB pathway

#### Components of non-canonical NF-κB pathway

Non-canonical NF-κB signaling activated by stimuli such as B lymphocyte activating factor (BAFF), CD40 ligand (CD40L), and lymphotoxin β (LTβ) does not require IKKβ or NEMO but instead relies on NF-κB-inducing kinase (NIK) and IKKα.^82^ NIK is a central component of non-canonical NF-κB signaling. The hallmark of non-canonical NF-κB signaling is the stabilization of NIK via ubiquitination and proteasomal degradation.^83,84^ NIK not only can activate IKKα but also facilitates binding between IKKα and p100, a process that is dependent on two amino acid residues of p100 (aa 866, 870).^85^ IKKα binds to p100 and phosphorylates serines 99, 108, 115, 123, and 872 on p100.^85^ p100 is subsequently ubiquitylated and partially degraded to active p52 by β-transducin repeats-containing proteins (β-TrCP) ubiquitin ligase and the 26 S proteasome.^8^ p100 also functions to inhibit RelB nuclear translocation.^83,86^

Non-canonical NF-κB signaling activated by receptors such as CD40, B lymphocyte stimulating factor receptor (BAFF-R), and lymphotoxin beta receptor (LTβR) involves the degradation of TRAF3, which is dependent on cIAP1/2 and TRAF2.^87^ IAP promotes the proteasomal degradation of NIK via the E3 ubiquitin ligase activity promotes proteasomal degradation of NIK, which can act as a regulator of NF-κB signaling.^88^ Activation of NF-κB signaling and TNF-α production by IAP antagonist compounds (IACs) was observed in tumor cell lines.^89^ The binding of TRAF2 to cIAP1/2 promotes TRAF2 and TRAF3 dimerization and recruitment of NIK.^90^ TRAF3 binds to the sequence motif ISIIAQA at the N-terminal end of NIK and promotes proteasomal degradation of NIK, thus acting as a negative regulator of NIK.^84^ NIK dissociates from the cIAP1/2-TRAF2 ubiquitin ligase complex and activates downstream IKKα.^87^

#### Activation and regulation of non-canonical NF-κB pathway

Most of the non-canonical NF-κB receptors belong to the TNFR superfamily, including BAFF-R, CD40, LTβR, and receptor Activator of NF-KappaB (RANK), which are associated with the recruitment of different TRAF members and bind to the corresponding ligands as complexes.^83^ TRAF members trigger the disassembly of the receptor-ligand complexes and further trigger the activation of NIK.

LT and tumor necrosis factor ligand superfamily member 14 (LIGHT) expressed in lymphocytes can act as ligands that bind to LTβR on the surface of lymphoid stromal cells and epithelial cells to activate NIK and mediate canonical and non-canonical NF-κB signaling by recruiting TRAF2/3/5.^83^ In canonical NF-κB signaling, LTβR promotes the expression of inflammatory genes such as macrophage inflammatory protein-1β (MIP-1β), MIP-2, and vascular cell adhesion molecule-1 (VCAM-1).^91^ In non-canonical NF-κB signaling, LTβ R mainly mediates B lymphocyte chemoattractant (BLC), EBI-1-ligand chemokine (ELC), secondary lymphoid tissue chemokine (SLC), stromal cell-derived factor-1 α (SLC), secondary lymphoid tissue chemokine (SDF-1α), and BAFF, and other genes related to secondary lymphoid organogenesis and homeostasis.^91^ BAFFR expressed in B cells preferentially induces the non-canonical NF-κB signaling pathway, which mediates B cell survival, development, and maturation.^83,92–94^ 73-75 BAFF-R binds more strongly and rapidly to TRAF3, a property that is primarily associated with the BAFF-R signaling motif PVPAT.^93^ Degradation of TRAF3 activates non-canonical NF-κB signaling, and induction of the canonical NF-κB pathway requires TRAF2.^83^ CD40 is primarily expressed in B cells, and upon binding to CD40L on the surface of activated T cells, one pathway activates non-canonical NF-κB signaling through the recruitment of TRAF2 and TRAF3, and the other pathway participates in canonical NF-κB signaling through the recruitment of TRAF6.^83^ CD40-activated non-canonical NF-κB signaling is mainly involved in the regulation of T-B cell interactions, B cell proliferation, survival, and antibody isotype switching.^95^ Receptor activator of NF-KappaB ligand (RANKL)/RANK interaction is not only involved in the regulation of osteoclast development and activation, but also mediates immune cell survival, communication, and lymphoid organ formation.^96^ RANK-activated non-canonical NF-κB signaling promotes osteoclastogenesis and differentiation.^97,98^

## Crosstalk of NF-κB signaling

**Fig. 2:**
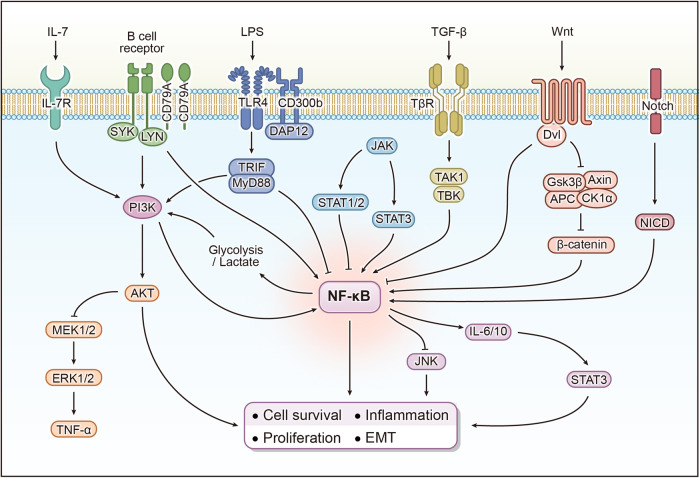
The crosstalk between NF-κB signaling and other signaling pathways. (1) The activation of PI3K by BCR and IL-7R via the cIAP-IKK pathway results in the stimulation of NF-κB. Hepatitis B virus X protein induces aerobic glycolysis and produces lactate through the NF-κB/hexokinase 2 pathway, activating the PI3K/AKT signal; (2) NF-κB inhibits TNF-α-mediated JNK signaling; (3) The product of NF-κB signaling, IL-6, can activate STAT3. JAK-STAT3 can act as an upstream regulator of NF-κB, promoting NF-κB signal transduction, while STAT1 inhibits NF-κB-mediated tumor cell survival; (4) TAK1 promotes NF-κB transcriptional activity; (5) Wnt/β-catenin signaling activates NF-κB in the cytoplasm. Dvl inhibits NF-κB signaling in the nucleus; (6) Notch1 binds to NF-κB, promoting NF-κB transcriptional activity. APC adenomatosis polyposis coli protein, CK1α casein kinase 1 alpha, DAP12 DNAX-activating protein of 12 kDa, Dvl disheveled, EMT epithelial-mesenchymal transition, ERK extracellular regulated protein kinase, GSK3β glycogen synthase kinase 3 beta, IL interleukin, IL-7R interleukin 7 receptor, JAK Janus kinase 2, JNK c-Jun N-terminal kinase, LPS lipopolysaccharide, LYN LYN proto-oncogene, Src family tyrosine kinase, MEK mitogen-activated protein kinase, MyD88 MYD88 innate immune signal transduction adapter, NF-κB nuclear factor kappa B, NICD Notch intracellular domain, PI3K phosphatidylinositol 3-kinase, STAT signal transducer and activator of transcription, SYK spleen associated tyrosine kinase, TAK1 TGF-beta activated kinase 1, TBK TANK-binding kinase, TGF transforming growth factor, TLR toll-like receptor, TNF tumor necrosis factorα, TRIF toll-like receptor adapter molecule 1, TβR TGF-beta receptor, Wnt wingless-type MMTV integration site family

Signaling molecules transmit regulatory signals intracellularly or extracellularly and act as receptors, ligands, protein kinases, or transcription factors in signaling pathways. The different signaling pathways constitute a signal transduction network with a fine-grained regulatory system through mutual interactions. NF-κB signaling is not isolated in the regulation of numerous physiological and pathological processes in which it is involved, and there may be direct or indirect regulation with other molecules, which in consequence, triggers interactions with other signaling pathways. Classical signaling pathways include NF-κB, PI3K/AKT, MAPK, JAK-STAT, TGF-β, Wnt, Notch, and Hedgehog signaling. These signaling pathways may interact with NF-κB signaling in the involvement of biological processes such as cell proliferation, differentiation, survival, death, development, immunity, inflammation, and tumorigenesis. In addition, members of the TLR receptor family are also engaged in NF-κB signaling by recognizing antigenic components of microorganisms. When placing vision in the sophisticated molecular regulatory network, it contributes to our better comprehension of NF-κB signaling by shedding light on its interactions with the abovementioned pathways (Fig. 2).

### Crosstalk of NF-κB signaling with PI3K/AKT signaling

The phosphatidylinositol 3-kinase (PI3K)/AKT signaling pathway, as a crucial cellular signaling pathway, is involved in the regulation of several biological activities, which include, but are not limited to, cellular metabolism, proliferation, survival, and angiogenesis, and is in turn involved in the regulation of oncology, metabolism, immunity, angiogenesis, and cardiovascular homeostasis.^99–101^ Signals from growth factors, cytokines, and cytokines bind to the cell surface receptor tyrosine kinase (RTK) or G protein-coupled receptor (GPCR) and promote PI3K-catalyzed production of phosphatidylinositol trisphosphate (PIP3), and PIP3 is the second messenger that activates AKT.^100,102^ Phosphatidylinositol-4,5-bisphosphate 3-kinase catalytic subunit alpha (PIK3CA) encodes the p110α catalytic subunit of PI3K, and its mutation is one of the most common somatic alterations in solid tumors.^103^

The interplay between NF-κB signaling and PI3K/AKT signaling in diffuse large B cell lymphoma (DLBCL) is a significant phenomenon. The proliferation and survival of activated B-cell type diffuse large B cell lymphoma (ABC-DLBCL) cells require active BCR signaling, and activation of NF-κB signaling is detected in ~10% of ABC-DLBCL, and the BCR-PI3K-NF-κB signaling cascade has been suggested as a potential target for the treatment of DLBCL as a potential target.^104,105^ Recent findings have revealed that PI3K activates NF-κB signaling through the cIAP-IKK pathway, and copanlisib, a dual inhibitor of PI3Kα/δ, may effectively block PI3K/AKT signaling and NF-κB signaling in ABC-DLBCL, leading to tumor regression.^106^ Inhibition of PI3Kβ/δ in DLBCL was also found to decrease NF-κB activity.^107^ The dual inhibitor of PI3K and HDAC, CUDC-907, also reduced the activity of AKT, p65, and BCL-XL in multiple myeloma (MM) in a dose-dependent manner.^108^ Another exemplary case is atherosclerosis. IL-7, which is essential for T cell development and balance, activates NF-κB signaling via the PI3K/AKT pathway, upregulates the expression of monocyte chemotactic protein 1 (MCP-1) and cell adhesion molecule (CAM) in macrophages and human aortic endothelial cells, and plays an active role in atherosclerosis.^109^ Increased secretion of the pro-inflammatory factor galectin-3 (Gal-3) in atherosclerosis activates the PI3K/AKT pathway and inhibits autophagy upon binding to CD98, whereas inhibition of Gal-3 reduces the activity of the NF-κB pathway, suppresses inflammation, and enhances autophagy.^110^

NF-κB signaling may also interact with PI3K/AKT signaling through metabolic pathways. In hepatitis B Virus (HBV)-related hepatocellular carcinoma (HCC), hepatitis B protein X (HBx) induces aerobic glycolysis and produces a large amount of lactic acid through NF-κB/hexokinase 2 (HK2) signaling, which further activates PI3K/AKT signaling and improves the malignant proliferation ability of HCC cells.^111^ Somatostatin receptor subtype 2 (sst2) inhibits KRAS-activated PI3K signaling. Studies in KRAS^G12D^, sst2± hybrid mice demonstrated that PI3K/AKT signaling activates NF-κB signaling and activates KRAS, promoting the release of CXC chemokine ligand 16 (CXCL16) and IL-6, ultimately leading to the progression of pancreatic ductal adenocarcinoma (PDAC).^112^ The PI3K/AKT/NF-ΚB signaling system also facilitates the epithelial-mesenchymal transition (EMT).^113^

### Crosstalk of NF-κB signaling with MAPK signaling

The mitogen-activated protein kinase (MAPK) belongs to the serine/threonine kinase family and plays an important role in diverse cellular programs such as proliferation, differentiation, development, transformation, inflammatory responses, and apoptosis by transmitting, amplifying, and integrating signals from a broad spectrum of stimuli. MAPK signaling is a conserved enzymatic cascade that mediate signal transduction from the cell surface to the nucleus through phosphorylation events. This pathway involves three key enzymes: mitogen-activated protein kinase kinase kinase (MAPKKK), mitogen-activated protein kinase kinase (MAPKK), and mitogen-activated protein kinase (MAPK). MAPK is responsible for phosphorylating target proteins in the cytoplasm or nucleus. MAPKs in mammalian cells mainly include extracellular regulated protein kinase (ERK), p38 MAPK, c-Jun N-terminal kinase (JNK), and extracellular regulated protein kinase 5 (ERK5). The transcriptional specificity of NF-κB can be achieved through interaction with the MAPK pathway.^8^ Evidence of NF-κB signaling’s interaction with MAPK signaling has primarily centered on JNK signaling. TAK1 serves as an upstream kinase for both NF-κB signaling and JNK signaling.^10^ The JNK pathway regulates cell cycle progression through multiple mechanisms. JNK activates c-Jun and activator protein-1 (AP-1) to exert pro-oncogenic effects, while simultaneously inducing apoptosis.^114^ Cellular responses exhibit variability based on the nature of the stimulus, the extent of JNK activation, and the duration of the response.^114^ Studies investigating the interaction of NF-κB signaling with JNK signaling have revealed that although JNK signaling regulates cell death or survival, the ultimate fate of the cell is determined by NF-κB, and activation of NF-κB signaling is capable of inhibiting pro-apoptosis induced by caspases, JNK, and reactive oxygen species (ROS).^115^ Negative regulation of TNF-α-mediated JNK signaling by NF-κB has been identified in murine embryonic fibroblasts, and it is important to note that this negative crosstalk is specific to TNF-α signaling.^116,117^ NF-κB was also observed to block TNF-induced apoptosis through the downregulation of JNK and c-Jun/AP-1 in rat hepatocytes.^118^ Sst2 also activates NF-κB signaling through Src homology region 2domain-containing phosphatase 1 (SHP-1), leading to the inhibition of JNK phosphorylation and apoptosis.^119^ During acute liver failure, interleukin 1 receptor type 1 (IL-1R1) is stimulated by IL-1 and activates the NF-κB signaling, which promotes transcriptional upregulation of inflammation-related genes and recruitment of immune cells, while NF-κB inhibits TNF-activated JNK/ERK signaling and prevents caspase 3-mediated apoptosis, which further amplifies inflammatory responses and exacerbates hepatic injury.^120^

### Crosstalk of NF-κB signaling with JAK-STAT signaling

Janus kinase 2 (JAK) binds non-covalently to cytokine receptors, mediates the tyrosine phosphorylation of the receptor, and recruits one or more signal transducer and activator of transcription (STAT) proteins. Upon phosphorylation, STAT proteins translocate across the nuclear membrane to modulate the activity of specific genes. The JAK family comprises JAK1, JAK2, JAK3, and tyrosine kinase 2 (TYK2).^121^ Erythropoietin mediates the activation of JAK2 in neurons, which further activates NF-κB signaling and initiates the transcription of genes with neuroprotective effects.^122^ The STAT family consists of STAT1, STAT2, STAT3, STAT4, STAT5A, STAT5B, and STAT6.^123^ Each STAT protein exerts unique biological effects and plays a regulatory function in cell survival, differentiation, metabolism, and immune response, and plays a key role in malignant tumors and autoimmune diseases.^124^ STAT1 helps boost immunity against tumors, yet STAT3 and other types of proteins may trigger pro-cancer inflammation.^125^ A close interaction between STAT3 and NF-κB signaling has been observed. IL-6, a gene product regulated by NF-κB signaling, is an important STAT3 activator.^126^ IL-10 and CpG synergistically activate STAT3 and NF-κB in a human B cell line induced by MYC.^127^ STAT3 also inhibited the expression of molecules essential for NF-κB and STAT1-mediated antitumor immunity, including IL-12 and interferon (IFN)-γ.^128,129^ STAT3-mediated acetylation of RelA promotes NF-κB to exert pro-transcriptional activity in the nucleus, a phenomenon observed in both tumor cells and tumor-associated hematopoietic cells.^130^ Deletion of Abelson interactor 1 (Abi-1) may lead to increased activity of STAT3 and NF-κB, which may be a potential mechanism leading to primary myelofibrosis.^131^ In colorectal cancer, IKKα induces the cytokine leukemia inhibitory factor (LIF) by inducing NF-κB dependent transcriptional activity, thereby activating STAT3.^132^ In NIK-positive anaplastic lymphoma kinase (ALK)-negative anaplastic large cell lymphoma cells, STAT3 promotes the expression of p52 and CD30, thereby inducing sustained activation of non-canonical NF-κB signaling.^133^ STAT3 promotes the degradation of p100 to p52 through the activation of IKKα. This process necessitates the activation of STAT3 by cyclic adenosine monophosphate (cAMP)-response element-binding protein (CREB)-binding protein (CBP)/p300.^134^ STAT3 not only promotes tumor cell proliferation, survival, neovascularization, and metastasis but also exerts an inhibitory effect on anticancer immunity.^125^ IFN-γ and TNFα promote the inducible nitric oxide synthase (iNos) gene promoter’s response to NF-κB through activation of JAK-STAT signaling in muscle fibroblasts recruitment, thereby activating the iNOS/nitric oxide (NO) pathway and inducing muscle atrophy.^135^

### Crosstalk of NF-κB signaling with TGF-β signaling

Members of the transforming growth factor (TGF)-β family include TGF-β, activating factor, and bone morphogenetic protein (BMP). These cytokines play crucial roles in diverse cellular processes, including cell proliferation, migration, metabolism, immune regulation, and inflammatory response.^136^ The TGF-β family of receptors comprises the type I receptor TGF-beta receptor (TβR) I, the type II receptor (TβRII), and the type III receptor (TβRIII), among which TβRI and TβRII possess intrinsic kinase activity, which is essential for TGF-β signaling. Upon binding to the ligand, TβRII phosphorylates the serine and threonine residues of TβRI. Activated TβRI subsequently phosphorylates the downstream signaling molecule Smad, leading to its nuclear accumulation and transcriptional regulation as a transcription factor. In the early stage of tumorigenesis, the TGF-β family exerts an oncogenic effect by inhibiting cell proliferation. However, as the tumor continues to progress, tumor cells develop resistance to TGF-β-mediated growth inhibition, which is attributed to mutations in genes encoding signaling intermediates.^137,138^ TCR inhibits TβRI expression and TGF-β signaling through activation of NF-κB signaling and CARMA1, resulting in the quiescence of T cells.^139^ Smad7 inhibits TNF signaling by forming a complex with TAB2 and TAB3, thereby suppressing NF-κB activation and inflammatory responses.^140^ However, NF-κB in glioblastoma activates TGF-β by inducing miR-148a or miR-182, leading to hyperactivation of both NF-κB and TGF-β signaling.^141,142^ TAK1 promotes the phosphorylation and transcriptional activity of NF-κB, which mediates inflammatory response, EMT, tumor metastasis, chemoresistance, etc.^143–145^ Whereas TAK1 exerts a negative regulatory effect on IKK in neutrophils after stimulation by LPS, which is in contrast to the previous perceptions.^146^ TGF-β induces ubiquitination degradation of MyD88 to negatively regulate pro-inflammatory signaling, specifically through the recruitment of Smad ubiquitination regulatory factor (Smurf) 1 and Smurf2 with E3 ubiquitin ligase activity by Smad6.^147^ TGF-β also stimulates cardiac inflammation and fibrosis through activation of NF-κB signaling.^148^

### Crosstalk of NF-κB signaling with Wnt signaling

The wingless-type MMTV integration site family (Wnt) signaling pathways encompass Wnt/β-catenin, Wnt/planner cell polarity (PCP), and Wnt/Ca2+ pathways. The Wnt/β-catenin signaling pathway is a β-catenin-dependent class of Wnt signaling, also known as the canonical pathway, which mainly controls cell proliferation. The Wnt/PCP and Wnt/Ca2+ pathways are not dependent on β-catenin and are known as non-canonical pathways that regulate cell polarity, adhesion, and migration. In the Wnt/β-catenin pathway, lipoprotein receptor-related protein (LRP) and frizzled (FZD) act as Wnt receptors and form a complex with Wnt proteins to activate downstream signaling. During the development of acute myocardial infarction, elevated Wnt2 promoted β-catenin/NF-κB signaling by binding to Fzd4 and LRP6, and elevated Wnt4 activated the same signaling by binding to Fzd2 and LRP6, resulting in a pro-fibrotic effect.^149^ Axis inhibition protein (Axin)/ adenomatosis polyposis coli protein (APC)/ glycogen synthase kinase 3 beta (GSK3β)/ casein kinase 1 alpha (CK1alpha) complex phosphorylates and inactivates β-catenin. NF-κB transcriptional activation is decreased in GSK3-deficient embryonic fibroblasts without affecting IκB degradation and nuclear translocation of NF-κB.^150^ Disheveled (Dvl) impedes the Axin/APC/GSK3β/CK1α complex in the cytoplasm, which inhibits the degradation of β-catenin and promotes its translocation to the nucleus, and activates proliferation- and differentiation-related genes by interacting with the T-Cell factor (TCF) family of transcription factors and activating coactivators.^151,152^ In contrast, it has been revealed that Dvl interacts with p65 in the nucleus and inhibits NF-κB-mediated transcriptional activation, and promotes apoptosis, independently of Wnt or β-catenin.^153^ β-TrCP, a ubiquitin E3 ligase, promotes ubiquitylated degradation of β-catenin in response to resting Wnt signaling. During endotoxemia, NF-κB and Wnt/β-catenin signaling are mutually activated, and β-TrCP mediates the degradation of IκB to upregulate NF-κB signaling. Activated NF-κB, in turn, promotes the production of Wnt, β-catenin, and β-TrCP, which leads to cytokine storms, liver injury, and even death.^154^ Wnt signaling may also interact with non-canonical NF-κB signaling. LTβR was found to inhibit WNT/β-catenin signaling in alveolar epithelial progenitor cells by activating non-canonical NF-κB signaling, thereby promoting lymphocyte apoptosis and inhibiting regeneration.^155^

### Crosstalk of NF-κB signaling with Notch signaling

The Notch signaling consists of Notch receptors, Notch ligands, CBF-1/Suppressor of hairless/Lag (CSL)-DNA-binding proteins, intracellular effector molecules, and regulators of Notch, which regulate diverse cellular processes including proliferation, stem cell maintenance, differentiation, and death.^156^ The classical NOTCH signaling does not necessitate amplification by a cascade of second messengers and protein kinases, and the receptor is directly transported to the nucleus after three cleavage events.^157^ The Notch receptor consists of an extracellular domain (NEC), a transmembrane fragment (NTC), and an intracellular domain (NTC). Notch intracellular domain (NICD).

When Notch signaling is transmitted in two neighboring cells, the Notch receptor interacts with the ligand and undergoes triple shearing, releasing the activated form of Notch, NCID, into the nucleus and binding to the transcription factor CSL to regulate downstream gene expression. The network of interactions between Notch and NF-κB may contribute to the pathogenesis of T-cell acute lymphoblastic leukemia.^158,159^ One possible mechanism is that the intracellular structural domain of Notch1 may compete with IκBα for binding to NF-κB and promote the transcriptional activity of NF-κB.^160^ It has also been found that Notch inhibits the deubiquitinase CYLD (a negative regulator of IKK) via HES1 to maintain NF-κB activity.^161^ In Barrett’s esophagus mouse model, Notch signaling activates NF-κB and regulates the differentiation of gastric cardia progenitor cells.^162^ Apurinic/apyrimidinic endonuclease (APE1) is activated in a variety of cancers and induces transcription of target genes by interacting with several redox-dependent transcription factors.^163,164^ APE1 promotes the activation of Notch signaling in esophageal adenocarcinoma through redox-dependent NF-κB activation and upregulation of delta-like protein 1 (DLL1) (Delta-type Notch ligand), which is critical for cancer cell stemness, inflammation, and embryonic development.^164^ In myeloproliferative disorders, the transcription of miR-155 is inhibited by Notch/RBPJ, leading to attenuated miR-155 inhibition of κB-Ras1 (an inhibitor of NF-κB), thereby promoting NF-κB signaling as well as the production of pro-inflammatory cytokines.^165^ The target gene of Notch signaling, HES1, represses Deltex1 transcription by binding directly to a site located 400 bp upstream of the Deltex1 transcriptional start site, thereby leading to the restoration of Notch1 expression.^166^ In medullary thyroid carcinoma with RET mutation, nuclear translocation of NF-κB binds to and enhances the expression of the miR-182 promoter, inhibits HES1 and upregulates Deltex1, ultimately promoting tumor invasion and migration.^167^ Overexpression of p52 and RELB in a mouse pluripotent stem cell line resulted in elevated levels of RBP and HES1, which were dependent on NICD.^168^ Notch is also an important upstream regulator of non-canonical NF-κB signaling, and it was found that γ-secretase inhibitor (GSI) XII inhibited Notch signaling in Hodgkin’s and Reed-Sternberg’s cells, further down-regulated the expression of p52 and RelB, and inhibited the conversion of p100 to its active form, p52.^169^

### Crosstalk of NF-κB signaling with Hedgehog signaling

Hedgehog signaling is a highly conserved pathway with important roles in the control of cell proliferation, tissue homeostasis, tumorigenesis, and embryonic development.^170,171^ Members of the Hedgehog gene family include Sonic Hedgehog (SHh), Indian Hedgehog (IHh), and Desert Hedgehog (DHh), of which SHh has been the most widely and intensively studied. In the resting state, Smoothened (Smo) is inhibited by Hedgehog’s receptor PTCH. When Hedgehog binds to PTCH, activated Smo transmits signals through Gli, Sufu, and Kif7, resulting in the generation of the Gli-activated form (GliA), which translocates to the nucleus and leads to transcriptional activation of Hh target genes.^170,171^ NF-κB signaling plays a pivotal role in the generation of apical ectodermal ridges of limb buds during development, and inhibition of NF-κB in vertebrate limb mesenchyme downregulates the expression of SHh and Twist.^172^ In chronically damaged fibrotic livers, Smo suppresses transcriptional expression of miR-378a-3p via p65 activation, which subsequently upregulates the expression level of Gli3.^173^ High expression of p65, SHh, and Gli1 was observed to be associated with poorer prognosis in patients with advanced prostate cancer. Experimental verification in cell lines observed inconsistent results, although NF-κB signaling and SHh-Gli1 signaling activation were observed in both PC3 and DU145 cell lines, whereas in PC3 cell lines, Gli1 activation was only dependent on SHh, while in DU145 cells, Gli1 expression was neither dependent on SHh nor NF-κB.^174^ Therefore, further investigation is required to elucidate the crosstalk mechanism between NF-κB and Hedgehog signaling.

### Crosstalk of NF-κB signaling with TLR signaling

Toll-like receptor (TLsR) is a single-channel transmembrane protein consisting of an extracellular region, a transmembrane region, and an intracellular region, and it belongs to pattern recognition receptors (PPRs). Mammalian TLRs are expressed in a number of cell types, including macrophages, dendritic cells, B cells, stromal cells, and epithelial cells. TLR recognizes and interacts with surface and intracellular components of microorganisms, activates innate immunity and mediates the development of acquired immunity.^175^ The TLR signaling pathway originates from a conserved intracellular structural domain of the receptor consisting of ~200 amino acids, the Toll/IL-1R (TIR) domain. TLR binding to ligands induces the formation of dimers or conformational changes that activate TLR signaling, recruit downstream signaling molecules, and ultimately lead to the activation of NF-κB and MAPK signaling, among others.^176^

However, TLR does not necessarily always mediate the activation of NF-κB signaling. There may be a negative regulatory relationship between the two under the influence of other molecules or pathways. A typical case is found in macrophages, where LPS inhibits NF-κB signaling by inducing Inducible cAMP early repressor (ICER) expression via p38-mediated cAMP response element-binding protein (CREB), a negative feedback loop that is an important mechanism for preventing TLR-driven excessive inflammation.^177^ The downstream kinase mitogen-and stress-activated protein kinase (MSK)1/2 of p38 phosphorylates and activates the transcription factor CREB, which promotes the transcription of related genes.^178^ Phosphorylated CREB inhibits NF-κB activation by competing with p65 for binding to CREB-binding protein (CBP).^179^ ICER is induced by CREB and constitutes a negative regulatory loop by binding and inhibiting the cAMP response element.^180^ Another prominent example pertains to CD300b, which functions biologically as a binding receptor for LPS. CD300b and its adapter, DAP12, activated splenic tyrosine kinase (Syk) and PI3K upon binding to LPS and TLR4, promoting the dissociation of MyD88-TIRAP, which further inhibited the activation of the MEK1/2-ERK1/2 and NF-κB pathways via AKT, thereby inhibiting the production of the anti-inflammatory factor IL-10 and driving the cytokine response and aggravating septic shock.^181^

## Physiology and pathology of NF-κB signaling

### Physiological roles of NF-κB signaling

**Fig. 3:**
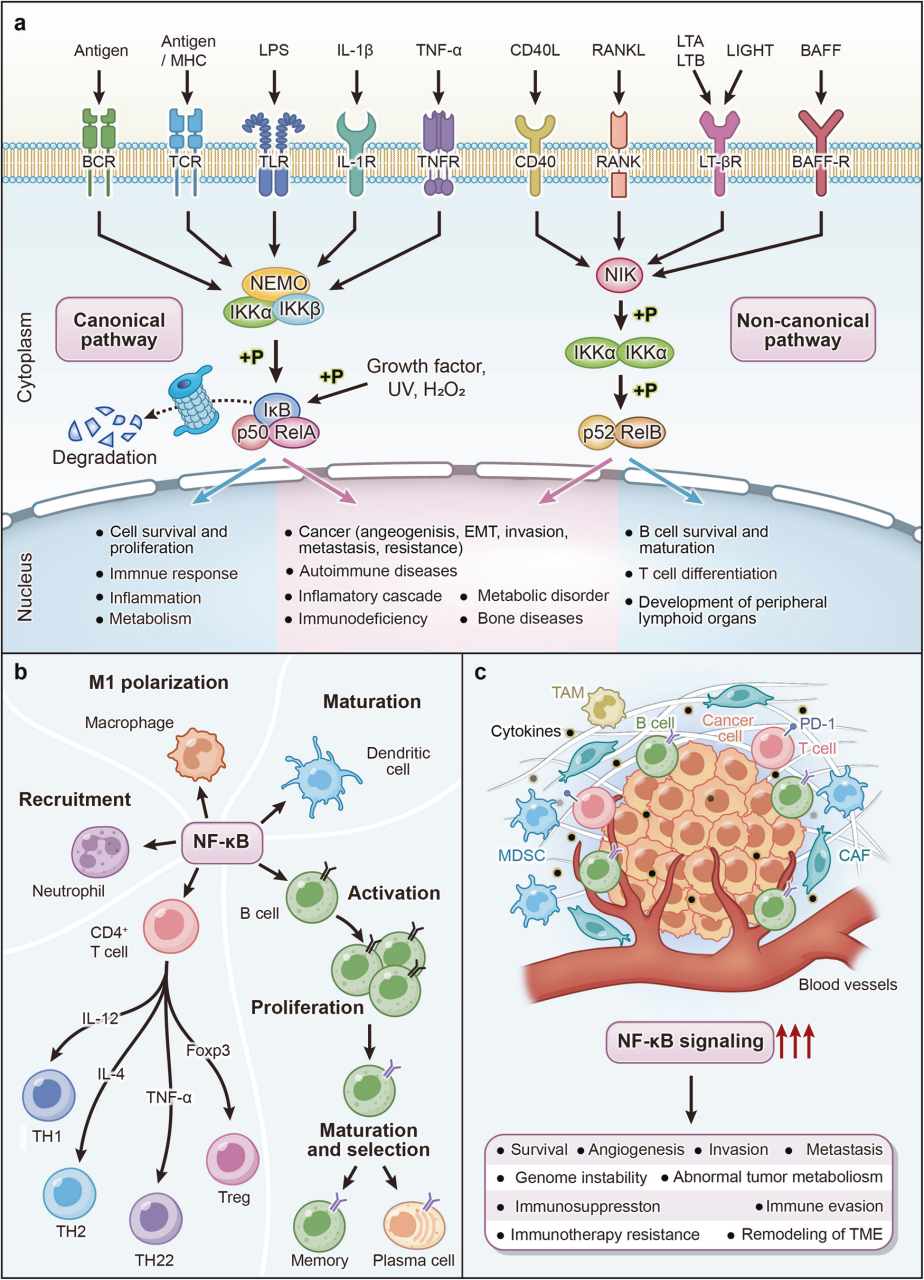
The biological functions of NF-κB signaling. **a** The NF-κB signaling supports cell survival under physiological settings, modulates inflammation and immunological responses to external stimuli, and also helps to regulate metabolism and homeostasis. Overactivation of the NF-Κb signaling increases tumor malignancy in pathological settings, including angiogenesis, EMT, invasion, metastasis, and treatment resistance. Furthermore, NF-κB signaling dysregulation can result in inflammatory storms and metabolic problems. BAFF TNF superfamily member 13b, BAFF-R TNF receptor superfamily member 13 C, BCR B-cell receptor, IKK I-kappaB kinase, IKK I-kappaB kinase, IκB IkappaB protein, LIGHT tumor necrosis factor ligand superfamily member 14, LPS lipopolysaccharide, LTA lymphotoxin alpha, LTB lymphotoxin beta, LT-βR lymphotoxin beta receptor, MHC major histocompatibility complex, NEMO inhibitor of nuclear factor kappa-B kinase subunit gamma, NIK mitogen-activated protein kinase kinase kinase 14, RANK TNF receptor superfamily member 11a, RANKL TNF superfamily member 11, TCR T-cell receptor, TLR toll-like receptor, TNF tumor necrosis factor, TNFR TNF receptor. **b** NF-κB plays a pivotal role in both innate and adaptive immunity. In the context of innate immunity, NF-κB promotes the differentiation of macrophages into M1 phenotype. Additionally, NF-κB facilitates dendritic cell maturation and neutrophil recruitment. Concerning adaptive immunity, NF-κB enhances the activation, proliferation, maturation, and selection of B cells. Moreover, under the stimulation of different cytokines, NF-κB can drive the differentiation of CD4 T cells into various subtypes. TH helper T cell, Treg regulatory T cell, IL-12 interleukin-12, TNF-α tumor necrosis factor-alpha, Foxp3 forkhead box protein 3. **c** Tumor occurrence and progression are closely linked to TME. Overactivation of NF-κB signaling not only promotes tumor cell survival, invasion, metastasis, genomic instability, and metabolic abnormalities, but also reshapes the immune-suppressive microenvironment, promoting immune escape and resistance to immunotherapy. CAF cancer-associated fibroblasts, EMT epithelial-mesenchymal transition, MDSC myeloid-derived suppressor cell, PD-1 programmed death 1, TAM tumor-associated macrophage, TME tumor microenvironment

NF-κB plays a key role in cellular responses to external stimuli such as cytokines, stress, UV light, antigens, and heavy metals. Existing studies have demonstrated that the NF-κB signaling is involved in a diverse array of physiological and pathological processes, including immune and inflammatory responses, cell survival and proliferation, metabolism, as well as synaptic plasticity and memory-related activities^81,182,183^ (Fig. 3a).

NF-κB signaling is particularly important in regulating cellular adaptation to environmental changes. In response to inflammatory stimuli, immune cells reconfigure metabolism through cellular responses mediated by NF-κB signaling. Drosophila studies revealed that NF-κB maintains the coordination of innate immune-metabolic responses by inhibiting Foxo-mediated lipolysis.^184^ Muscle contraction involves activation of NF-κB signaling by Ca^2+^, peroxides, and nitrogen oxides.^185–187^ It has been demonstrated that NF-κB signaling is activated during the strenuous exercise of the organism, either in normoxia or acute hypoxia, which includes the increase of p105, p50, IKKα, IκBβ, and glutathione reductase protein levels as well as CaMKII δD phosphorylation. When exercise ends and the muscle resumes open circulation, these changes return. The design of the new study needs to take into account the rapid changes in NF-κB signaling during exercise cessation.^188^

TLR-induced NF-κB activation upregulates the transcription of genes encoding inflammatory vesicles and initiates immune responses.^189^ Inflammation serves as a pivotal defense mechanism against bacterial and viral infections. Serine/threonine kinase 4 (Stk4) and NF-κB are involved in the activation and homeostasis of regulatory T (Treg) cells and promote Treg cell-mediated immune tolerance. Deletion of Stk4 in mouse Treg cells inhibits p65 expression, p65-Foxp3 complex formation, and Treg cell activation, ultimately leading to autoimmune lymphoproliferative disorders.^190^ The IKK complex protects mature T cells from TNF-induced cell death and is important for their normal homeostasis and function.^7^ The integrity of cellular function requires rapid activation and termination of NF-κB signaling, and this tight regulation is essential for normal cellular and organismal homeostasis.^191^ N6-methyladenosine (m6A) mRNA modification is involved in the maintenance of colonic epithelial cells and stem cell homeostasis. Studies in mouse colon epithelial cells have revealed that methyltransferase 14 (Mettl14) inhibits colonic epithelial cell apoptosis by modulating the NF-κB pathway.^192^

### Pathological roles of NF-κB signaling

The pathological effects of NF-κB signaling include immune disorders, malignant behavior of tumor cells, metabolic dysregulation, and skeletal disorders. These effects are further described below.

Due to the key regulatory role of NF-κB signaling in immune and inflammatory responses, its dysregulation has been strongly associated with a variety of human diseases, including cancer, inflammatory diseases, autoimmune disorders, viral infections, and infectious shock.^81,189,193^ During inflammation, the NF-κB signaling is hyperactivated, leading to the abundant expression of inflammation-associated genes. Initially, researchers discovered that NF-κB potentially contributes to the pathogenesis of acquired immune deficiency syndrome (AIDS) by synergizing with and stimulating the transcription of human immunodeficiency virus (HIV).^194^ Research on p50-deficient mice has demonstrated the crucial involvement of NF-κB in both specific and non-specific immune responses, and although there is no evidence for the involvement of NF-κB in the developmental process.^195^

As a chronic ailment, the prevalence and fatality of neoplasms persistently escalate, posing a significant peril to human existence and well-being. NF-κB signaling is involved in tumorigenesis, progression, EMT, tumor metastasis, and drug resistance.^6,191^ NF-κB signaling is a major pathway mediating the interaction between inflammation and cancer. As a result of alterations in the inflammatory microenvironment and oncogenic mutations, sustained NF-κB activation and dysregulation of cellular functions are observed in cancer, leading to genomic instability and gene mutations, creating a microenvironment that promotes tumor progression and promotes proliferation and angiogenesis of tumor cells while inhibiting their apoptosis.^6,191,196^

Recent research has unveiled the pivotal role of NF-κB in the cellular response of tumors to nutrient-deprived microenvironments, and the main mechanism is to remodel the local metabolism by coordinating the actions of glycolysis, glutaminolysis, and oxidative phosphorylation pathways.^184,197–201^ Impairment of canonical and non-canonical NF-κB signaling may lead to specific developmental and immune deficiencies.^202,203^ For instance, germline mutations in NFKB2 in non-canonical NF-κB signaling affect the nuclear translocation of p52, which is thought to be the genetic cause of primary immunodeficiency syndromes.^204^

The non-canonical NF-κB pathway is crucial in lymphoid organ development, lymphocyte survival and homeostasis, dendritic cell activation, osteoclastogenesis, etc., and its aberrant activation may lead to rheumatoid arthritis, ulcerative colitis, osteoporosis, and lymphoid malignancies.^83,205–208^ Expression of RelB subunits is associated with the differentiation of dendritic cells and thymic UEA-1+ medullary epithelial cells, which provides the basis for its involvement in immune responses.^209^ NF-κB receptor activator ligand (RANKL), an osteoclast differentiation factor, has an influential role in osteoclastogenesis, linking the activated immune system to bone loss.^210–213^

### NF-κB signaling, immune system, and inflammation

The NF-κB family is a crucial component of both innate and adaptive immunity, and plays a vital role in immune response regulation. Upon stimulation by various inducers, NF-κB undergoes translocation to the nucleus, where it binds to specific DNA sites and orchestrates the transcriptional control of numerous genes. These genes encompass antimicrobial peptides, cytokines, chemokines, stress response proteins, and anti-apoptotic proteins, among others.^79^ Persistent activation of the NF-κB pathway is frequently implicated in inflammatory conditions like rheumatoid arthritis, inflammatory bowel disease, multiple sclerosis (MS), and asthma.^214^ Gaining deeper insights into the modulation of the NF-κB pathway holds the potential to establish targeted therapies for inflammatory diseases. In this section, we will delve into the interplay between the NF-κB signaling pathway and the immune system, spanning both innate and adaptive immunity (Fig. 3b).

#### Innate immunity

Innate immune cells, such as macrophages, dendritic cells, and neutrophils, play a critical role in innate immunity and the inflammatory response. These cells express pattern recognition receptors (PRRs) that are capable of detecting a wide range of microbial components known as pathogen-associated molecular patterns (PAMPs).^215,216^ Additionally, PRRs are also involved in recognizing molecules called damage-associated molecular patterns (DAMPs), which are released by necrotic cells and damaged tissues.

One crucial signaling pathway activated by PRRs is the canonical NF-κB pathway, which plays a significant role in the induction of pro-inflammatory cytokines, chemokines, and other inflammatory mediators in various innate immune cell types. These inflammatory mediators can directly contribute to inflammation or indirectly promote the differentiation of inflammatory T cells.

One common signaling transduction event of pattern recognition receptors (PRRs) is the activation of the canonical NF-κB pathway, which is responsible for the transcriptional induction of pro-inflammatory cytokines, chemokines, and other inflammatory mediators in different types of innate immune cells.^217^ The process of PRRs activating the NF-κB pathway is as follows: downstream of PRRs, LPS/TLR4 converges through myd88-dependent and TRIF-dependent signaling pathways to activate IKK via TRAFs. The dsRNA/RIG-I signal is transmitted to IKKi/TBK1 through ISP1 and then to IKK through RIP1. The signaling from NOD to NF-κB is believed to involve RIP2 oligomerization and the induction of proximity to activate IKK.^218^ Intestinal epithelial cells (IECs) express various PRRs, including TLRs, on their basolateral and apical cell membranes. When encountering microbial ligands, these receptors initiate cascades of signaling events leading to the activation of NF-κB and other pro-inflammatory pathways.^219,220^ Additionally, NF-κB serves as a central mediator for the activation initiation signal of the NLRP3 inflammasome, responding to various PRR ligands and cytokines by inducing the transcriptional expression of NLRP3 and pro-IL-1β.^221^

#### Adaptive immunity

Adaptive immunity is a specific immune response by the body against particular antigens, mainly mediated by T and B lymphocytes. NF-κB regulates the functions of multiple immune cells in adaptive immunity through gene transcription regulation. First, NF-κB participates in regulating T cell development and activation.^222^ Under normal conditions, most T cells are in a resting state, but when stimulated, NF-κB is activated and enters the cell nucleus, promoting the transcription of specific genes, thus initiating T cell proliferation and differentiation processes.^223^ Furthermore, NF-κB also regulates B cell development and function. Upon antigen stimulation, NF-κB is activated in B cells, inducing their proliferation and differentiation.^224^ NF-κB is also involved in regulating antibody class switching and affinity maturation in B cells.^225^ These processes are crucial for the formation of specific antibodies and memory responses in the body.

In addition to regulating T and B cell development and activation, NF-κB also controls the expression of pro-inflammatory cytokines in adaptive immunity.^225^ When immune cells are infected or damaged, NF-κB is activated and induces the synthesis of various pro-inflammatory cytokines, such as tumor necrosis factor-alpha (TNF-α),^226^ interleukin-1 beta (IL-1β),^227^ and interleukin-6 (IL-6).^228^ These cytokines can trigger inflammatory reactions and attract other immune cells to eliminate pathogens or repair damaged tissues. Moreover, NF-κB also plays an important role in immune regulation in adaptive immunity. It participates in regulating immune tolerance and immune suppression. Some immune suppressive cells, such as regulatory T cells (Tregs),^229^ can inhibit the activity of other immune cells by activating the NF-κB pathway, maintaining immune balance and self-tolerance.

In conclusion, NF-κB plays a crucial role in adaptive immunity. It regulates T and B cell development, activation, and function, and is involved in antibody class switching and immunological memory formation. Additionally, it controls the expression of pro-inflammatory cytokines and immune regulatory processes. Further research into the mechanisms and regulatory networks of NF-κB will contribute to a better understanding of the regulatory mechanisms in adaptive immunity and may provide guidance for the development of novel immunotherapeutic strategies.

### NF-κB signaling and tumor microenvironment

The tumor microenvironment comprises immune cells, fibroblasts, myeloid-derived inflammatory cells, signaling molecules, surrounding vasculature, and the extracellular matrix (ECM), which constitutes an interacting population with tumor cells and plays an integral role in tumorigenesis and malignant progression.^230^ Tumor-associated macrophages (TAMs) represent the predominant immune cell population within the tumor microenvironment. They engage in complex interactions with tumor cells, T cells, endothelial cells, and fibroblasts, which can either promote immune evasion, tumor growth, and invasion, or exert antitumor effects.^231,232^ IL-1β produced by IFN-γ-polarized TAM promotes PIM2 expression in hepatocellular carcinoma cells through MAPK signaling and NF-κB signaling, conferring the ability of tumor cells to metastasize, immune escape, and resist immunotherapy.^233^ Tumor-associated macrophages (TAMs) and cancer-associated fibroblasts (CAFs) have been shown to facilitate tumor angiogenesis through the secretion of proangiogenic and pro-inflammatory factors.^234^ Platelets activate TGF-β/Smad and NF-κB signaling in tumor cells during intravascular transit from the primary tumor to the metastatic site, promoting tumor metastasis and EMT.^235^ Histamine secreted by glioblastoma stem cells triggers the activation of endothelial cells by the Ca^2+^-NF-κB axis, remodeling the tumor microenvironment and thereby promoting angiogenesis and tumor progression.^236^ CXCL2 and CXCL8 generated in tumor-infiltrating monocytes via the 6-phosphofructo-2-kinase/fructose-2,6-bis-phosphatase3 (PFKFB3)-NF-κB axis promotes neutrophil recruitment in the hepatocellular tumor microenvironment.^237^ The tumor microenvironment may shape gene expression and cellular phenotypes of immune cells or tumor cells, and this evolutionary change is the result of cellular adaptation under selective pressure.^238,239^ Activation of aryl hydrocarbon receptor (AHR) in TAMs by glioblastoma-produced kynurenine recruits CCL2 and inhibits activation of NF-κB signaling.^240^ Polynutrients activate NF-κB in cancer cells, leading to changes in cytokine production, neutrophil recruitment, and the immunosuppressive microenvironment to promote metastasis.^241^ Due to the chronic inflammatory state within the tumor microenvironment, myeloid-derived suppressor cells (MDSCs) are generated and activated to exert immunosuppressive functions.^242^ Cysteine-rich intestinal protein 1 (CRIP1) activates NF-κB signaling and upregulates CXCL1/5 expression to recruit MDSCs, leading to an immunosuppressive environment in pancreatic ductal adenocarcinoma.^243^ Tumor cells also interact metabolically with stromal cells in the tumor microenvironment.^189^ NIK may act as a key regulator of antitumor immunity and T-cell metabolism, and its deficiency impairs aerobic glycolysis and suppresses CD8+ effector T-cell function^201^ (Fig. 3c).

Tumor-induced chronic inflammatory microenvironments may lead to immunosuppression and promote immune escape.^191^ Overexpression of cell cycle-related kinase (CCRK) in chronic liver disease activates NF-κB signaling, promotes CXC motif chemokine ligand (CXCL)1 expression in polymorphonuclear-myeloid-derived suppressor cells (PMN-MDSC), and remodels the immunosuppressive microenvironment to resist metastasis-associated immune surveillance.^244^ In addition to solid tumors, NF-κB signaling has also been implicated in the microenvironment of hematologic tumors, and it has been demonstrated that NF-κB drives pro-survival, genetic instability, and immune evasion in refractory or relapsed diffuse large B-cell lymphoma.^245^

Although immunotherapy may induce durable responses in cancer patients, it inevitably faces the same challenges of drug resistance as chemotherapy, targeted therapy, and other therapies.^246,247^ CD10 + GPR77 + CAF, defined by specific cell surface markers, promotes tumor progression and chemoresistance through sustained activation of NF-κB signaling and complement signaling and secretion of IL-8 and IL-65 to maintain stemness of tumor stem cells.^248^ Inactivating mutations in TRAF3, TRAF2, CYLD, and cIAP1/2 lead to persistent activation of non-canonical NF-κB signaling.^206^ The extracellular matrix, comprising fibronectin, glycosaminoglycans, proteoglycans, and mucus, is a dynamic collaborator of the immune system. Immune cells can directly manipulate the synthesis and catabolism of the basic components of the ECM, or they may indirectly regulate the ECM through the secretion of cytokines.^249^ The study on triple-negative breast cancer has revealed that the molecular and physical properties of the ECM may exert varying impacts on treatment response. The ECM of untreated tumors is thought to be a hard microenvironment, whereas a soft ECM enhances drug resistance by increasing NF-κB signaling activity and downregulating pro-apoptotic JNK signaling activity.^250^ The tumor microenvironment may be one of the culprits for therapeutic resistance, and researchers have found that maintaining or inhibiting the expression of certain molecules in the tumor microenvironment is a pathway for overcoming drug resistance.

## NF-κB signaling in human diseases

### Cancers

**Fig. 4:**
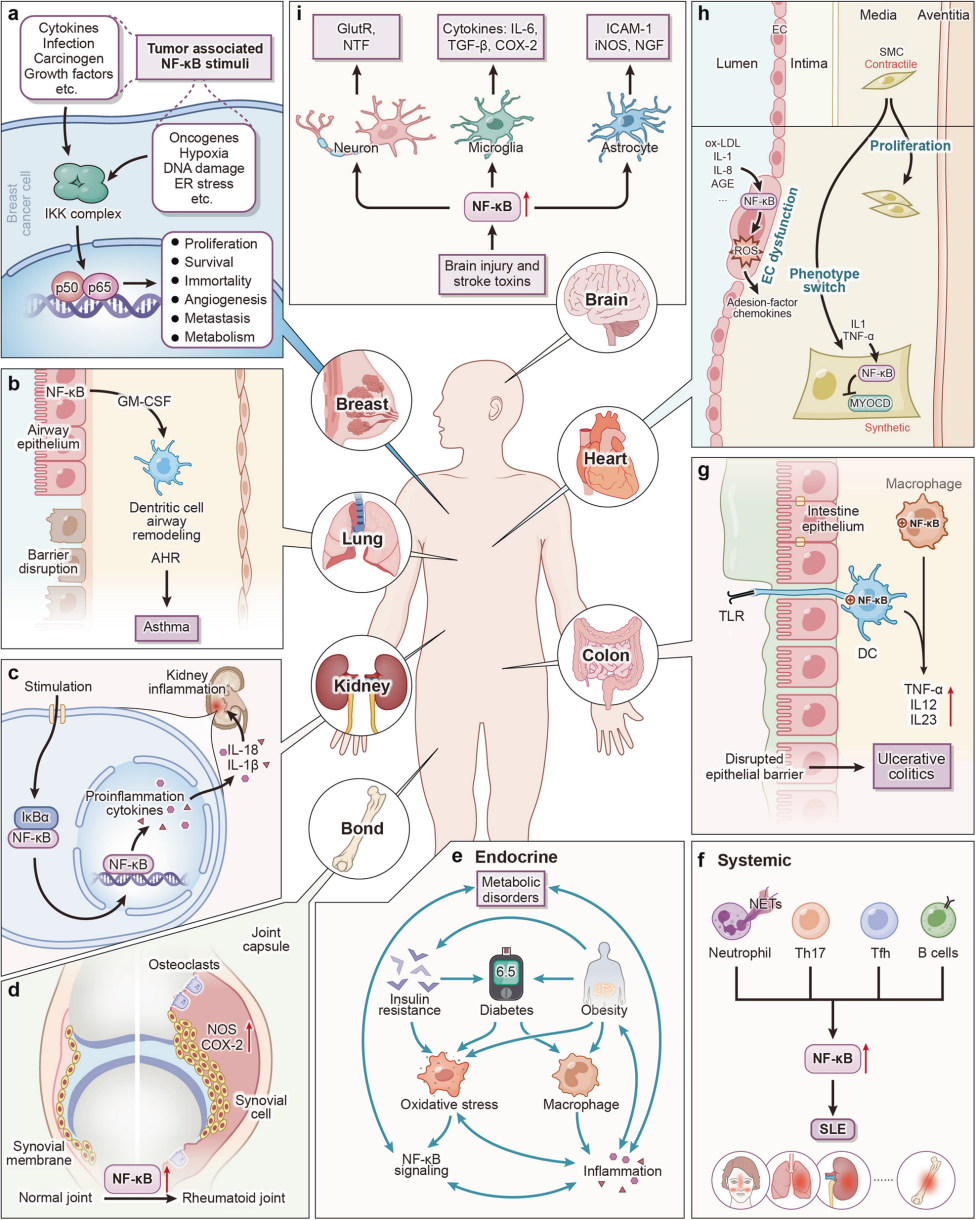
NF-κB plays a crucial role in diseases affecting various organs and systems. **a** NF-κB is also upregulated in breast cancer cells, leading to increased downstream gene expression promoting tumor growth, metastasis, and angiogenesis. **b** Increased expression of NF-κB in respiratory epithelial cells exacerbates TH2 cell-related inflammatory responses and airway hyperresponsiveness, so leading to asthma. **c** In the kidney, activated NF-κB promotes high expression of inflammatory factors IL-1β and IL-18, leading to renal inflammation. **d** Activated NF-κB promotes chronic inflammation, fibroblast-like synoviocyte proliferation, and thus contributes to the development of RA in synovial tissues. **e** There exists a bidirectional relationship between NF-κB signaling, metabolic diseases, and inflammation. Metabolic diseases like insulin resistance, diabetes, and obesity can cause overactivation of NF-κB signaling and inflammation through the regulation of oxidative stress and macrophage function. **f** NF-κB also facilitates the activation of polyclonal B cells and the production of autoantibodies in patients with SLE. **g** In macrophages, NF-κB activation induces the secretion of pro-inflammatory cytokines, including TNF-α, IL-12, and IL-23, which directly or indirectly participate in the mucosal tissue damage typically observed in UC. **h** NF-κB modulate a series of inflammatory mediators and thus participates in the regulation of different cell fates in the atherosclerotic process. **I** Following brain injury, NF-κB is upregulated in neurons, astrocytes, and microglial cells, resulting in the secretion of more inflammatory factors such as IL-6 and iNOS, thereby triggering local brain inflammation. TBK1 serves as a protective factor by suppressing NF-κB signaling. AGE advanced glycation endproducts, AHR airway hyper reactivity, COX-2 cyclooxygenase-2, DC dendritic cell, EC endothelial cell, ER estrogen receptor, FasL factor-related apoptosis ligand, GlutR glutamyl-tRNA reductase, GM-CSF granulocyte-macrophage colony-stimulating factor, ICAM-1 intercellular cell adhesion molecule-1, iNOS inductible nitric oxide synthase, MMP9 matrix metalloproteinase-9, MN-SOD manganese superoxide dismutase, MYOCD myocardin, NETs neutrophil extracellular traps, NGF nerve growth factor, NTF neurotrophic factor, ox-LDL oxidized low-density lipoprotein, RA rheumatoid arthritis, ROS reactive oxygen species, SLE systemic lupus erythematosus, SMC smooth muscle cell, TBK1 TANK-binding kinase 1, TGF-β transforming growth factor-β, TLR4 toll-like receptor 4, UC ulcerative colitis

In normal cells, NF-κB is kept inactive in the cytoplasm by binding with IκB. Upon degradation of IκB, NF-κB translocates into the nucleus to activate target genes and carry out its biological functions. Constitutive activation of NF-κB has been implicated in various solid tumors together with hematological tumors (Fig. 4a).

NF-κB can be activated by a wide range of inducers, including both extrinsic stimuli (cytokines, viral and bacterial products, carcinogens, etc.) and intrinsic stimuli (cellular stress, DNA damage, hypoxia, oncogene activation, etc.). The target genes transcriptionally regulated by NF-κB modify the gene expression pattern in cells to cope with the changes and threats faced by the organism. However, these responses can be highly pleiotropic and the outcomes of NF-κB activation largely depend on the context. Although the targets of NF-κB in tumor cells may be similar to those in normal cells, the negative feedback control is dysregulated in cancers, leading to sustained inhibition or activation of target genes.^251^ The effects of aberrant NF-κB include activating proto-oncogenes and genes involved in cell-cycle to promote tumor proliferation, inhibiting apoptosis to support the survival of cancer cells, regulating genes related to cell adhesion to facilitate metastasis. Additionally, NF-κB has a critical role in the metabolic reprogramming of cancer cells, promoting adaptive response to metabolic stress and thus contribute to tumor progression (Table 1).

**Table 1:** Overview of the mechanisms of NF-κB signaling in human diseases and main references

Disease	Mechanism	Reference
**A. Cancers**		
Proliferation	Induce the expression of cyclins and proto-oncogenes	^252–256,615,616^
Apoptosis	Depend on the balance between apoptotic and survival proteins	^258–262^
Angiogenesis	Upregulation of proangiogenic factors	^263,264,266–273,617^
Metastasis	Promote EMT, facilitate cancer extravasation and colonization	^276–282^
Immune evasion	Foster an immunosuppressive TME	^243,262,283,284^
Metabolic reprogramming	Remodeling of cellular metabolism, facilitate metabolic adaptation to nutrient deprivation.	^189,197–200,286–291^
Drug resistance	Regulate pro- and anti-apoptotic factors, metabolic reprogramming, gut microbiota, gene mutation	^191,292,294,295,297–301^
**B. Inflammation and autoimmune diseases**		
Rheumatoid arthritis	Promotes the proliferation of FLS, activation of immune cells, cytokine production	^313–318^
Osteoarthritis	Cartilage destruction, chondrocyte apoptosis	^325–328^
Multiple sclerosis	Formation of the local inflammatory microenvironment	^309,332–337^
Ulcerative colitis	Secretion of pro-inflammatory cytokines	^341–344^
Systemic lupus erythematosus	Promote the proliferation of T cells and B cells	^346–348,351,352^
Gluten induced enteropathy (Celiac Disease)	Secretion of pro-inflammatory cytokines, oxidative stress	^356–358^
Gout/Hyperuricemia	Systemic metabolic disturbances	^360–364^
Periodontitis	Secretion of pro-inflammatory cytokines, degradation of the bone matrix	^367–369^
Sepsis	Inflammatory responses	^372–375^
Asthma	Initiation and maintenance of asthmatic allergic inflammation	^378–382^
Kidney inflammation and injury	Secretion of pro-inflammatory cytokines	^384–390^
**C. Cardiovascular diseases**		
Atherosclerosis	Dysfunction and inflammation of ECs, foam cell formation,	^396,401,403–407,409^
Myocardial infarction	Secretion of pro-inflammatory cytokines	^411^
**D. Metabolic disorders**		
Insulin resistance and diabetes	Secretion of pro-inflammatory cytokines, oxidative stress	^182,429–437,439–441^
Obesity	Chronic low-grade inflammation	^424,445–448,451–455^
**E. Nervous system diseases**		
Parkinson’s disease	Secretion of pro-inflammatory cytokines	^458,460–464^
Brain injury	Upregulation of numerous immune mediators	^466^
Spinal cord injury	Inflammatory responses	^471,472^
**F. Corona virus disease 2019**	Secretion of pro-inflammatory cytokines, inflammatory cascades	^474–478^

#### Proliferation

NF-κB plays a pivotal role in tumor proliferation and progression. Major mechanisms include inducing the expression of cyclins and proto-oncogenes. Early in 1999, Guttridge et al revealed that NF-κB controls cell growth and differentiation through transcriptionally regulating cyclin D1 using both skeletal muscle differentiation models and normal diploid fibroblasts.^252^ More recent studies focusing on cancer showed that PAK upregulation enhanced cyclin D1 through NF-κB in breast cancer, consequently coordinating cell-cycle movement.^253^ Mutations in driver genes are also shown to have a close relationship with the NF-κB pathway in cancer cells. Gain-of-function mutations in oncogenes (such as RAS superfamily) or positive regulators (such as NF-κB inducing kinase) contribute to sustained activation of NF-κB signaling and subsequent cell proliferation. Xia et al. reported increased nuclear translocation of NF-κB was observed in K-rasG12D mutated mice, while IKKβ depletion or NF-κB signaling inhibition impairs lung adenocarcinoma development. Vreka et al. further confirmed that IKKα interacts with mutated KRAS and is necessary for the initiation and progression of KRAS-mutated lung adenocarcinoma.^254^ Furthermore, the dual roles of non-coding RNAs have also been reported in NF-κB-mediated tumor growth. Zhou et al., found that galectin-3 can activate TLR4 signaling and promote NF-κB translocation through the induction of lncRNA-NEAT1 (nuclear enriched abundant transcript 1) to facilitate lung adenocarcinoma cell proliferation.^255^ MicroRNAs, particularly miR-505, has also been reported to inhibit lung cancer proliferation through AKT/NF-κB pathway.^256^

#### Apoptosis

NF-κB has a dual role in the regulation of apoptosis, which is dependent on the balance between genes that controls cell survival and apoptosis.^257^ In cancer cells, NF-κB activity interacts with various apoptotic and survival proteins. NF-κB can regulate PTEN through transcriptionally activate Snail, a repressor of PTEN, and thus regulate cell survival.^258^ Man et al. reported a regulatory loop in the bladder cancer that overexpression of miR-130b/301b induced by NF-κB decreased USP13 expression and thus downregulate PTEN, which also facilitated the full activation of NF-κB.^259^ Lee et al., reported the bidirectional regulation of TRAIL, which promotes apoptosis via the ERK2/NF-κB signaling pathway in neuroepithelioma.^260^ YM155, a survivin inhibitor, can potentiate TRAIL-mediated apoptosis through inhibiting Mcl-1, c-FLIP, and NF-κB in breast cancer.^261^ Additionally, NF-κB can transcriptionally activate the Bcl-2 family, which inhibits BAX/BAK and thus prevents cytochrome C release and apoptosome formation. Furthermore, NF-κB modulates p53-mediated apoptosis via promoting the polyubiquitylation and degradation of p53.^262^ These findings mainly reflect its role in facilitating tumor resistance to apoptosis during tumor development.

#### Angiogenesis

Inducing angiogenesis is an essential hallmark of cancer. Growth factors are major regulators of angiogenesis, including vascular endothelial growth factor (VEGF), fibroblast growth factor (FGF), and platelet-derived growth factor (PDGF). These proangiogenic factors can be transcriptionally regulated by NF-κB in cancer, and the inhibition of NF-κB has been proven to prevent tumor angiogenesis in various cancer models, such as ovarian cancer, renal cell carcinoma, breast cancer, and colorectal cancer.^263–266^ Another widely investigated mechanism is its modulation of adhesion molecules in the formation of new blood vessels, such as intercellular adhesion molecule 1 (ICAM-1) and vascular cell adhesion molecule 1 (VCAM-1) on endothelial cells.^267–269^ Additionally, the activation of NF-κB in immune cells and tumor-associated macrophages stimulates the release of pro-inflammatory cytokines, such as TNF-α and interleukin-6 (IL-6), which further enhance tumor angiogenesis. NF-κB also induces the expression of hypoxia-inducible factor-1 alpha (HIF-1α), which is a master regulator of genes involved in angiogenesis.^270,271^ Some recent studies have deepened our understanding of its role in tumor angiogenesis. For example, Herkenne et al. reported that mitochondria-shaping protein OPA1 is required in an NF-κB-dependent signaling essential for developmental and tumor angiogenesis, which revealed the role of NF-κB in mitochondrial dynamics during angiogenesis.^272^ It is noteworthy that matrix metalloproteinases (MMPs) are also targets of NF-κB, which promote angiogenesis and metastasis in different microenvironments.^273^

#### Metastasis

The American Cancer Society estimates that there will be 1,958,310 new cases of cancer and 609,820 cancer-related deaths in 2023.^274^ Metastasis is responsible for 66.7% of solid tumor-related deaths.^275^ Epithelial-mesenchymal transition (EMT) is an essential event in tumor metastasis. NF-κB regulates an array of EMT-associated genes, including TWIST1, SNAIL, CDH2, etc.^276–279^ Li et al. reported the role of TNF-α/NF-κB/TWIST1 signaling axis in EMT in breast cancer, suggesting that targeting NF-κB-mediated Twist1 upregulation may be an effective therapeutic approach for breast cancer.^276^ Nomura et al. reported that, in the classical lymphovascular metastatic cascade, inhibition of NF-κB by specific inhibitor decreased the expression of several EMT transcription factors (SNAI1, SNAI2, and ZEB1) and mesenchymal markers (VIM and CDH2) and prevented in vitro invasion in pancreatic cancer, which can be rescued by IKK activation.^278^ A more recent study used human-derived metastasis models of renal cancer to identify transcriptional enhancers responsible for metastases. Researchers functionally characterized a coregulatory enhancer cluster, which was activated by HIF2A and an NF-κB-driven lymphoid element, as a mediator of metastasis in vivo.^279^ Recent studies also revealed the interaction between metastasis-associated proteins and NF-κB during EMT. El-Nikhely et al. reported the bidirectional role of metastasis-associated protein 2 (MTA2) in IKK2/ NF-κB-driven lung cancer progression.^280^ They found that metastasis-associated protein 2 (MTA2)/nucleosome remodeling and deacetylase (NuRD) corepressor complex can downregulate NF-κB signaling and inhibit tumor growth in an IKK2-independent manner. However, when MTA2/NuRD complex dissociates from the promoter region of NF-κB target genes and IKK2-dependent positive regulation of MTA2 occurs, it leads to the activation of NF-κB signaling and further promotes EMT and tumor metastasis. Cell adhesion molecules such as selectins and integrins are also largely modulated via the NF-κB pathway,^281^ which facilitates cancer extravasation and colonization. It is worthy of note that NF-κB also plays a role in shaping the pre-metastatic niche. Hiratsuka et al reported that inflammation mediator serum amyloid A3 (SAA3)/TLR4 signaling stimulates NF-κB activity in both lung epithelial cells and myeloid cells to establish an inflammatory state that facilitates metastasis.^282^

#### Immune evasion

NF-κB regulates tumor microenvironment (TME) to avoid immune surveillance and promote cancer progression in various aspects. The production of cytokines and growth factors regulated via NF-κB is significant in the establishment of immunosuppressive TME. For example, TGF-β increases the expression of the transcriptional coactivator MRTF-A in non-small-cell lung cancer (NSCLC) cells. Subsequently, MRTF-A interacts with NF-κB/p65 and facilitates the binding of NF-κB/p65 to the PD-L1 promoter. The activation of PD-L1 leads to immune evasion by NSCLC cells. The downregulation of MRTF-A in vivo can effectively inhibit lung tumor progression and enhance antitumor immunity.^283^

The role of NF-κB was not only implicated in cancer cells, but also in surrounding immune cells, such as CAFs, TAMs, and MDSCs. Inhibition of NF-κB signaling in CAFs abolished its tumor-promoting effects, suggesting the critical role of NF-κB in CAFs-mediated protumor effects.^262^ A single-cell RNA sequencing analysis suggested that the pattern recognition receptor Mincle was highly expressed in TAM, largely induced in bone marrow-derived macrophages by cancer cells to promote tumor development. Researchers reported a novel Mincle/Syk/NF-κB signaling circuit in TAM, which was a requisite for maintaining TLR4-independent protumoral activities. The blockade of Mincle/Syk/NF-κB signaling can repress the TAM-driven NSCLC progression in vivo.^284^ A recent study demonstrated that CRIP1 facilitated MDSC trafficking and fostered an immunosuppressive TME via facilitating NF-κB/p65 nuclear translocation in pancreatic ductal adenocarcinoma (PDAC), and inhibiting CRIP1/NF-κB/CXCL axis can sensitize PDAC to immunotherapy.^243^

#### Metabolic reprogramming

The emerging role of metabolic reprogramming has been demonstrated in the tumor progression,^285^ featuring high demand for nitrogen and deregulated mitochondrial oxidative phosphorylation. Remodeling of cellular metabolism via the NF-κB pathway can be addressed in various aspects.

RelA was a key component in sustaining mitochondrial oxidative phosphorylation, while its regulation of cellular metabolism depends on p53 status. In wild-type p53 colon carcinoma cells, RelA upregulates cytochrome c oxidase (synthesized by SCO2) to support oxidative phosphorylation. The inhibition of RelA leads to high vulnerability to glucose starvation and a large reduction in mitochondrial gene expression.^286^ However, in p53-deficient tumor, RelA interacts with heat shock protein mortalin and translocates to the mitochondria, thus repressing oxidative phosphorylation and cellular ATP levels.^287^

Additionally, the NF-κB pathway is critical in the metabolic adaptation to nutrient-depleted microenvironments. NF-κB transcriptionally regulates a wide array of metabolic enzymes, such as 6-phosphofructo-2-kinase/fructose-2,6-biphosphatase isoform 3 (PFKFB3), glutamate dehydrogenase 1 (GDH1), and carboxylesterase 1 (CES1), glutamine-fructose-6-phosphate transaminase 2 (GFPT2),^189,197–199,288^ facilitating metabolic adaptation to nutrient deprivation. For example, Reid et al. discovered a novel role of IKKβ in sensing glutamine-deprived environment and promoting metabolic adaptation. They found that IKKβ can directly interact with PFKFB3 at Ser269 upon glutamine deprivation to inhibit its activity, and thus downregulate aerobic glycolysis when glutamine levels are low.^197^

Recent studies have revealed a novel aspect of NF-κB in the cross-coordination of the immune response and metabolic systems. Researchers have reported that germinal center (GC) B cell-specific Rel loss in mice downregulate an array of metabolic genes, including genes involved in glycolysis and fatty acid oxidation, and therefore damage the formation of GCs.^289^ Rel also regulates an innate immune checkpoint that governs the function and metabolism of MDSCs and can promote oncogenesis via inhibiting antitumor immune responses. Rel suppression might enhance the therapeutic efficacy of current immune checkpoint immunotherapies.^200,290^ Infiltration of immunosuppressive macrophages is one of the defining characteristics of a pre-metastatic niche, which is a requisite for tumor metastasis. Morrissey et al. reported a novel role of NF-κB in promoting the immunosuppressive phenotype of macrophages through metabolic reprogramming. Tumor-derived exosomes (TDEs) increased glucose uptake through TLR2/NF-κB signaling and inhibits mitochondrial oxidative phosphorylation. This leads to elevated conversion to lactate, which feeds back on NF-κB and subsequently drives PD-L1 expression.^291^ These findings underlined the pivotal role of the NF-κB pathway in linking metabolic remodulations with inflammasome-driven cellular responses in cancer.

#### Drug resistance

Chemotherapy and radiotherapy largely act by inducing apoptosis in proliferating cells. However, as mentioned above, the constitutive activation of NF-κB signaling leads to evasion of apoptosis, by regulating a series of pro- and anti-apoptotic genes such as caspase-8 and c-FLIP, BCL-2, and BCL-XL.^191,292^ In some cases, chemotherapy and radiotherapy can activate NF-κB signaling, leading to acquired treatment resistance.^293^ For example, commonly used chemotherapy agents such as paclitaxel, cisplatin, gemcitabine, adriamycin, and vinblastine can activate the NF-κB cascade. Metabolic reprogramming and oncogenic signaling were significantly activated in doxorubicin-induced DLBCL-resistant cells. Further studies revealed that drug-resistant cells enhanced glycolysis through sustained activation of non-canonical NF-κB signaling, thereby promoting their survival. Targeting p52-RelB increased sensitivity to doxorubicin.^294^ The gut microbiota may be involved in chemotherapy resistance in malignant tumors. It has been found in a study that enrichment of Mycobacterium avium upregulated intratumoral LPS, which subsequently promoted prostate cancer progression and docetaxel resistance through activation of the NF-κB-IL6-STAT3 signaling axis.^295^ Tamoxifen is a commonly used endocrine medication for breast cancer, and the ensuing problem of drug resistance should not be ignored. Kotaro Azuma et al. found that tripartite motif-containing (TRIM) 47 could be a predictor of breast cancer recurrence through the analysis of 116 clinical samples of tamoxifen-treated breast cancer. Mechanistic studies found that TRIM47 promotes breast cancer proliferation and endocrine therapy resistance by forming a stable complex with protein kinase Cε (PKC-ε) and protein kinase D3 (PKD3) and activating NF-κB signaling.^296^ NF-κB signaling may also be implicated in the initiation and progression of castration-resistant prostate cancer (CRPCa), where it possibly relates to mutations, accumulation, and hypoactivity of the androgen receptor (AR).^297^ A genome-wide CRISPR-Cas9 screen performed in glioblastoma (GBM) identified E2F6 as the driver of temozolomide (TMZ) resistance, while EGFRvIII/ NF-κB is the upstream gene controlling E2F6 expression.^298^ Blockade of NF-κB can be a therapeutic target to reverse treatment resistance and enhance the effectiveness of anticancer treatments, which has been shown in numerous preclinical studies.^299–301^

### Inflammation and autoimmune diseases

#### General introduction

In order to gain a better understanding of the role of NF-κB in inflammation and autoimmune diseases, it is imperative to first comprehend its function in various immune cells.

##### Cells of innate immunity

Macrophages have been extensively studied regarding their pro-inflammatory function mediated by NF-κB. Upon recognition of diverse PAMPs and DAMPs, macrophages rapidly activate and release substantial amounts of cytokines. Depending on specific conditions, activated macrophages can differentiate into two distinct phenotypes: classically activated (M1) and alternatively activated (M2) macrophages. NF-κB serves as a key transcription factor for M1 macrophages, regulating an array of genes involved in inflammation, including TNF-α, IL-1β, IL-6, IL-12, and cyclooxygenase-2.^302^ Neutrophils and dendritic cells also play essential roles in local inflammation, with NF-κB being critical for their survival and function under potentially toxic conditions. This contributes to neutrophil recruitment and dendritic cell maturation.^303,304^

##### T cells

The NF-κB signaling pathway significantly influences the activation, differentiation, proliferation, and function of T cells. Upon binding of the antigen-MHC complex and CD80 or CD86 to the TCR and CD28, respectively, NF-κB complexes containing p65 are activated, leading to delayed and sustained activation of the c-Rel complex. NF-κB activity is crucial for activated T cells as it protects against apoptosis and promotes cytokine production, particularly IL-2, which supports proliferation and differentiation.^305,306^

NF-κB also plays a pivotal role in the differentiation of activated T cells into effector cells including Th1, Th2, Th17, and Treg cells. This process relies on the induction of specific transcription factors, including T-bet, GATA3, RORγt, and Foxp3. Increasing evidence suggests that NF-κB family members are key regulators of these processes. For instance, studies have shown that the absence of Foxo3a results in overactivation of NF-κB and increased production of TH1 and TH2 cytokines, leading to hyperactivated immunity.^307^ Additionally, mice lacking the p50 subunit of NF-κB fail to develop airway eosinophilic inflammation, indicating the critical role of NF-κB in Th2 differentiation.^308^ The TCR/CARMA1/NF-κB axis selectively drives Th17 differentiation through mechanisms involving cell cycle progression.^309^ Furthermore, TCR signaling induces nuclear translocation of serine/threonine kinase 4 (Stk4), resulting in the formation of the Stk4-Foxp3-NF-κB p65 complex, which regulates Foxp3 and p65-dependent transcription programs, thereby promoting Treg cell activation.^190^

##### B cells

Regarding B cells, the development and survival of immature B cells largely depend on NF-κB signaling downstream of BCR and BAFF. Throughout B cell development, the canonical NF-κB pathway activated by BCR regulates central tolerance, survival, and differentiation. Tonic BCR and BAFF stimulation contribute to the maintenance of naive B cells through NF-κB activation. BAFF signaling coordinates the activities of RelB and cRel to ensure survival during peripheral B cell maturation.^310^ In the germinal center, CD40L signals cooperate with BCR signaling to induce c-Myc expression through the NF-κB pathway, promoting B cell survival and re-entry into the cell cycle.^311^ Subsequently, NF-κB signaling in B cells expressing specific BCR leads to class switch recombination, resulting in the differentiation of B cells into either memory B cells or plasma cells.^289^ In summary, NF-κB plays a crucial role in supporting B cell proliferation, differentiation, and survival.

#### Rheumatoid arthritis

Rheumatoid arthritis (RA) is a chronic systemic autoimmune disorder typified by inflammation of the synovial joints. Its main pathological features include inflammatory proliferation of synovial tissue, formation of pannus, and erosion and destruction of articular cartilage and surrounding tissues.^312^ Although the exact pathogenesis of RA remains unclear, the role of NF-κB in its development has gained increasing attention with advancements in related research. Activated NF-κB promotes chronic inflammation and excessive proliferation of fibroblast-like synoviocytes (FLS) in the synovial tissue, thereby contributing to the progression of RA.^313^

NF-κB activation perpetuates chronic inflammation by targeting genes involved in inflammation during the progression of RA. NF-κB is involved in signaling transduction, activation, differentiation, and production of IFN-γ and IL-17 in inflammatory T cells, which are crucial for sustaining rheumatoid synovial inflammation.^314^ Additionally, the proliferation of B cells and the production of autoantibodies are closely associated with activated NF-κB members.^315^ Regarding innate immune regulation, dysregulation of NF-κB activation in dendritic cells can induce the production of cytokines such as IL-15 and IL-18, promoting the differentiation of inflammatory T cells.^316,317^ NF-κB also influences non-immune cells, as evidenced by increased expression of p50 and p65 in FLS cells of RA compared to normal synovium.^318^ NF-κB enhances the expression of cyclin D1 and c-Myc, positively regulating the cell cycle in fibroblasts and myoblasts.^319^ Moreover, NF-κB activation transmits anti-apoptotic signals in FLS through the induction of c-IAP.^320,321^ These repetitive cycles driven by NF-κB contribute to the worsening of the disease.

#### Osteoarthritis

Osteoarthritis (OA) is a degenerative disease characterized by the progressive deterioration of joints, with joint pain as the main clinical manifestation.^322^ The NF-κB pathway actively participates in and regulates various aspects of chondrocyte function, including proliferation, differentiation, apoptosis, and bone matrix metabolism.^323^ NF-κB plays a crucial role in the onset and progression of OA^323^ (Fig. 4d).

The NF-κB pathway can be activated by different ligands, such as tumor necrosis factor-alpha (TNFα).^324^ Upon activation, NF-κB translocates to the nucleus, leading to the transcription of downstream target genes, including matrix metalloproteinases (MMPs), a disintegrin and metalloproteinase with thrombospondin motifs (ADAMTS), and hypoxia-inducible factor 2-alpha (HIF2α).^325–327^ MMPs are known to degrade the extracellular matrix of articular cartilage, contributing to its breakdown.^325^ Furthermore, NF-κB promotes tissue inflammation and the release of catabolic factors by inducing chondrocyte apoptosis and facilitating the synthesis of prostaglandin E2 (PGE2), nitric oxide synthase (NOS), NO, and cyclooxygenase-2 (COX-2).^328^ These processes ultimately exacerbate joint damage in OA.

#### Multiple sclerosis

Multiple sclerosis (MS) is considered an immune disease primarily caused by the infiltration of peripheral immune cells into the central nervous system.^329^ NF-κB plays a significant role not only as a mediator of the inflammatory process in peripheral immune cells but also in microglia and astrocytes, making it important in the progression of MS.^330^

The differentiation of T cells into regulatory T cells (Tregs) requires the NF-κB subunit c-Rel, indicating that NF-κB could be a potential therapeutic target for MS.^190,331^ Furthermore, antigen-presenting cells (APCs) depend on c-Rel to produce IL-12 and IL-23, two cytokines that promote the differentiation of Th0 into Th1 or Th17 cells, respectively.^309^ Th17 cells secrete granulocyte-macrophage colony-stimulating factor (GM-CSF), which plays a crucial role in promoting the progression of MS.^332^ The activation of the NF-κB pathway is required for astrocytes to create a local inflammatory environment by inducing the secretion of inflammatory cytokines and chemokines.^333^ This inflammatory response can result in local tissue damage and attract additional inflammatory cells.^333^ Conversely, inhibiting the NF-κB pathway in astrocytes leads to the recruitment of CD8 + CD122+ regulatory T cells, thereby alleviating MS.^334–336^ Similarly, the activation of NF-κB in microglia or infiltrating macrophages may also influence the production of inflammatory cytokines and chemokine.^333,337^

#### Ulcerative colitis

Ulcerative colitis (UC) is a persistent inflammatory disorder affecting the mucosal lining of the colon that primarily affects the rectum and extends through part or all of the colon in a continuous manner.^338,339^ The pathogenesis of UC involves dysregulation of cytokine production and signaling mechanisms in intestinal epithelial cells, lymphocytes, and macrophages, with the transcription factor NF-κB playing a significant regulatory role in this complex process^340^ (Fig. 4g).

NF-κB activation in macrophages can induce the secretion of pro-inflammatory cytokines such as TNF-α, IL-1, and IL-6. NF-κB also regulates the expression of IL-12 and IL-23,^341,342^ which are involved directly or indirectly in the mucosal tissue damage commonly observed in inflammatory bowel disease (IBD). The activation of NF-κB by IL-6 in colon epithelial cells may contribute to increased expression of intercellular adhesion molecule-1 (ICAM-1), an important mediator in the recruitment of neutrophils to inflammatory sites.^343^ Furthermore, NF-κB activation in intrinsic intestinal fibroblasts leads to increased expression of cytokines like IL-8, IL-6, and monocyte chemoattractant protein.^344^ This suggests that colonic fibroblasts can participate in the immunopathogenesis of IBD in an NF-κB-dependent manner.

#### Systemic lupus erythematosus

Systemic lupus erythematosus (SLE) is a systemic autoimmune disease characterized by dysregulation of B cells and T cells, leading to the activation of polyclonal B cells and the production of autoantibodies.^345^ Nuclear NF-κB plays a significant role in the pathogenesis of SLE by promoting the proliferation of T cells and B cells^346–348^ (Fig. 4f).

In the context of SLE, the inflammatory environment can lead to the release of IL-6 or TNF-α, which activate the NF-κB pathway.^349^ This activation enhances the activity of the miR-34a promoter, resulting in the downregulation of Foxp3 expression and excessive proliferation of T cells.^350^ Additionally, NF-κB has been shown to be upregulated in autoreactive B cells mediated by CD40 and BAFF, promoting the secretion of autoantibodies.^351,352^ Furthermore, IFN-1 is a key factor in the pathogenesis of SLE, and its induction is mediated by NF-κB activation through TLR7 stimulation.^353^

#### Gluten induced enteropathy (Celiac disease)

Celiac disease (CD) is an immune-mediated intestinal disorder caused by intolerance to gluten ingestion.^354^ The pathogenesis of CD involves innate and adaptive immunity, primarily mediated by the infiltration of lymphocytes, particularly T cells, into the small intestinal epithelium.^355^ The upregulation of the NF-κB pathway and its downstream cytokines, such as IL-8, in the intestinal mucosa suggests the involvement of NF-κB in the development of celiac disease.^356^

In CD, various subtypes of T cells are activated, leading to the concurrent activation of different signaling cascades, including NF-κB, GATA1, JAK, and others.^357^ Gliadin peptides found in gluten induce changes in the oxidative balance of intestinal cells and are associated with the activation of the transcription factor NF-κB.^357^ NF-κB activation triggers the transcription of pro-inflammatory cytokines and enzymes like COX-2 and iNOS, leading to increased production of prostaglandins and NO metabolites, which contribute to oxidative stress. Recent studies have identified the m6A- exportin-1 (XPO1)-NF-κB axis as a potential target for CD treatment. Individuals carrying the XPO1 risk allele express higher levels of XPO1 protein, which results in increased downstream NF-κB activity and subsequent inflammation.^358^

#### Gout/Hyperuricemia

Gout is a chronic metabolic disorder characterized by the accumulation of sodium urate crystals in joints and non-articular structures.^359^ Sodium urate has been shown to possess pro-inflammatory activity. NF-κB is a key regulatory factor involved in controlling the production of inflammatory markers and mediators. In the context of gout, NF-κB activation induced by sodium urate contributes to systemic metabolic disturbances.^360^

Crystalline sodium urate acts as a damage-associated molecule that triggers innate immune pathways. For instance, sodium urate can stimulate NF-κB through TLR4 and TLR2, leading to the synthesis of pro-IL-1β and components of the inflammasome.^361^ Furthermore, sodium urate can induce renal inflammation in gouty nephropathy by activating tubular NF-κB signaling.^362^ Recent studies have also demonstrated the involvement of the pro-inflammatory NF-κB pathway in hypothalamic inflammation associated with metabolic syndrome.^363,364^

#### Periodontitis

Periodontitis is characterized by the pathological loss of periodontal ligament and alveolar bone.^365^ The disease involves complex interactions between specific bacterial pathogens and destructive immune responses.^365,366^ In a model of periodontitis, a single intra-gingival injection of LPS results in the significant expression of NF-κB p65 and pro-inflammatory cytokines in gingival tissues after 14 h. This is accompanied by increased expression of COX-2 and iNOS, as well as elevated levels of cytokines TNF-α and IL-1β. These changes are often associated with heightened nociceptive perception, supporting the involvement of NF-κB in the pathogenesis of periodontitis.^367^

Periodontitis is caused by the accumulation of microbial biofilms, with LPS being a major virulence factor present in the cell walls of these bacteria. LPS stimulates TLRs on microbial cells, resulting in the activation of NF-κB and subsequent upregulation of downstream inflammatory cytokines such as IL-1, IL-6, IL-8, and TNF-α.^368^ Furthermore, periodontal fibroblasts, when exposed to both TLR activation and mechanical force stimulation, activate the p38, JNK, and NF-κB pathways. This leads to the secretion of MMPs and degradation of the bone matrix.^369^

#### Sepsis

Sepsis is a systemic inflammatory response syndrome caused by the invasion of pathogenic microorganisms, predominantly bacteria.^370^ In critically ill patients with sepsis, multiple organ failure often occurs in addition to the manifestations of systemic inflammatory response syndrome and the primary site of infection.^371^ The pathophysiology of sepsis and septic shock involves a complex network of cytokines and inflammatory mediators.^372^ Central to this network is the activation of NF-κB.^373^ Studies have revealed that increased accumulation of nuclear NF-κB in peripheral blood mononuclear cells (PBMCs) of sepsis patients is associated with higher mortality rates and poorer clinical prognosis.^374^

Researchers have discovered that macrophages exhibit enhanced expression of vascular endothelial growth factor receptor 3 (VEGFR-3) in response to bacterial infection or stimulation with LPS. VEGFR-3 forms a negative feedback loop that inhibits TLR4-NF-κB-mediated inflammatory responses, thereby reducing the occurrence of sepsis or endotoxic shock resulting from bacterial infections.^375^ Inhibition of NF-κB activation has been shown to reduce acute inflammatory processes and organ dysfunction.^374^ These findings highlight the potential effectiveness of targeting NF-κB for the treatment of sepsis.

#### Asthma

Asthma is characterized by variable airflow obstruction, typically accompanied by bronchial constriction and airway inflammation resulting from the contraction or hypertrophy of airway smooth muscle (ASM).^376^ Multiple pieces of evidence suggest that the NF-κB pathway is upregulated in asthmatic tissues.^377^ PBMCs from adult patients with uncontrolled severe and moderate asthma exhibit higher levels of NF-κB p65 protein expression compared to individuals without asthma.^378^ This suggests that NF-κB plays a pivotal role in the initiation and maintenance of asthmatic allergic inflammation^379^ (Fig. 4b).

Previous studies have shown that activation of the TLR4 pathway by ovalbumin (OVA), an allergen commonly used to induce experimental asthma, leads to the upregulation of Th2-related inflammatory responses and promotes the gene expression of inflammatory cytokines, which is mediated by NF-κB.^380^ NF-κB, as a multi-cellular transcription factor, also plays a major role in regulating inflammation and immune responses by modulating Th2 cell cytokine production and gene expression.^381^ Studies using mice lacking the NF-κB p50 subunit have demonstrated reduced eosinophilic response to inhaled allergens. This effect is attributed to the lack of secretion of Th2 cell cytokines, including IL-13, IL-4, and the eosinophil growth factor IL-5, by T cells.^382^

#### Kidney inflammation and injury

Nephritis is an immune-mediated disease characterized by the formation of immune complexes resulting from antigen-antibody binding. These immune complexes deposit in various parts of the kidney, leading to pathological damage.^383^ IgA nephropathy, the most common form of glomerulonephritis, involves the binding of IgA to Fcα receptors on mesangial cells, which activates NF-κB and contributes to the induction of chemokines MCP-1 and IL-8.^384,385^ NF-κB also plays a role in lupus nephritis. Increased expression and activation of NF-κB have been observed in glomerular endothelial cells and mesangial cells of patients with lupus nephritis, accompanied by upregulation of inflammatory cytokines.^386^ NF-κB is critical in regulating autoimmune nephritis involving T cells and B cells. The Th17+ T cell subset, implicated in the pathogenesis of renal inflammation, requires the involvement of the classical NF-κB pathway for the generation of Th17 cells from naive T cells^387,388^ (Fig. 4c).

Acute kidney injury (AKI) is commonly caused by ischemia-reperfusion, during which the kidneys experience hypoxia and reduced blood flow. Inflammation induced by AKI contributes significantly to kidney injury, and controlling inflammation has shown effectiveness in reducing kidney damage and promoting recovery.^389^ NF-κB is activated during ischemia-reperfusion-induced renal injury and is considered a key mediator of inflammation. Studies have demonstrated that NF-κB inhibitors can attenuate renal inflammation and reduce injury induction in animal models.^390^

### Cardiovascular diseases

#### Atherosclerosis

Atherosclerosis is a chronic inflammatory disorder characterized by the aggregation of lipid particles in the arterial wall, the involvement of different cell types, and the activation of several important signaling pathways. Among them, the prominent role of NF-κB signaling in different stages of atherosclerosis is established.^391^ In fact, NF-κB signaling is a key element in the common pathogenesis of several atherosclerosis-associated diseases, including abdominal aortic aneurysm, peripheral artery disease, cerebrovascular disease and coronary artery disease.^392–395^ Endothelial cells (ECs) dysfunction plays a key role in the initiation of atherosclerosis. In response to pro-atherogenic factors including oxidized low-density lipoprotein (ox-LDL), IL-1, ROS and advanced glycation endproducts (AGEs), vascular ECs facilitate NF-κB activation and induces the expression of cytokine (such as IL-6 and TNF-α) and adhesion molecules (such as E-selectin, VCAM-1, ICAM-1).^396^ The process above promotes dysfunction and inflammation of ECs, and promoting the recruitment of monocytes and lymphocytes from the vascular lumen to the subendothelial layer of arterial intima. Furthermore, disturbed flow-induced ECs dysfunction through mechanotransduction pathways result in the activation of NF-κB signaling.^397^ Vascular smooth muscle cells (VSMCs) are the primary source of plaque cells.^398^ The major pathogenic mechanism underlying the formation of atherosclerotic plaques was believed to be the phenotypic switching of VSMCs from contractile to proliferative synthetic types in response to arterial inflammation.^399,400^ The synthetic state has also been linked to the activation of VSMCs proliferation and migration. Activated NF-κB components have been identified in VSMCs derived from human atherosclerotic lesions. Production of inflammatory cytokines such as IL-1 and TNF alter VSMCs phenotype via NF-κB mediated downregulation of contraction-relation gene expression.^401^ Additionally, phenotype switching, proliferation, and migration of VSMCs produced by PDGF-BB are prevented by disrupting the ROS/NF-κB signaling pathway.^402^ NF-κB also work in monocyte differentiation, macrophages polarization, maintenance, and transformation to foam cell. Ox-LDL engage receptor CD36 on macrophages and triggers TLR activation and excessive lipid accumulation through NF-κB activation, leading to foam cell formation in atherosclerotic plaques.^403,404^ Smad3/NF-κB pathways has been found to be involved in the transition between M1 and M2 subtypes in macrophages.^405^ Overexpressing of matrix metalloproteinase-9 (MMP-9)by macrophages promote the dissolution of plaque elastin and induces plaque disruption, which is associated with the activation of the TLR4/NF-κB pathway.^406,407^ In the end stage of atherosclerotic plaques, apoptosis of various cells becomes critical in controlling plaque stabilization.^408^ In human carotid plaques, NF-κB activation and factor-related apoptosis ligand (FasL)and active caspase-3 expression were analyzed and found to increase significantly compared to healthy controls.^409^ The underlying pathway regarding NF-κB induced apoptosis may be through the promotion of FasL via its receptor CD95 (Fig. 4h).

#### Myocardial infarction

The majority of myocardial infarction are the result of rupture of atherosclerotic plaques in blood vessels, followed by an abnormal coagulation reaction and formation of blood clot. Spontaneous or therapeutic revascularization leads to reperfusion of infarcted myocardium, which is called as reperfusion injury.^410^ Reperfusion injury triggers an inflammatory response, causing infiltration of inflammatory cells and excessive activation of myofibroblasts and vascular endothelial cells. The process above finally causes cardiac repair and cardiac fibrosis. In the cycle of ischemia and repair, TLR/NF-κB pathway has been widely researched and thought to be a critical role. Necrotic and damaged cells induced activation of TLR (especially TLR4), facilitate IκB kinase phosphorylation through MyD88/TAK1 pathway and finally engage NF-κB p65 and p50, leads to activation of plenty of inflammatory factors, including cytokines, chemokines, adhesion molecules and complement factor B.^411^ Inhibition of NF-κB has been proved to be associated with decreased reperfusion injury after myocardial infarction.^412–414^

### Metabolic disorders

At the core of metabolic homeostasis is the maintenance of normal nutrient perception and regulation by the body, and the interplay of the immune and metabolic systems is an important mechanism for this.^415^ The metabolic challenges encountered by contemporary humans are predominantly attributed to overnutrition rather than nutritional inadequacies. Metabolic disorders frequently exhibit interplay, and individuals with obesity are predisposed to developing type 2 diabetes (Fig. 4e).

#### Insulin resistance and diabetes

Insulin resistance, characterized by reduced sensitivity of the body to insulin, is a prevalent feature of metabolic disorders such as type 2 diabetes mellitus, dyslipidemia, and other metabolic disorders.^416^ It also serves as a common pathophysiological mechanism in diseases such as cardiovascular and cerebrovascular diseases, polycystic ovary syndrome, and tumors. Insulin is a protein hormone secreted by pancreatic β-cells in the pancreas in response to endogenous or exogenous stimuli. Insulin is the sole hormone in the body that possesses hypoglycemic properties and governs the energy metabolism of the liver, skeletal muscle, and adipose tissue. When insulin resistance occurs, glucose utilization decreases while hepatic glycogen breakdown into glucose increases, resulting in elevated blood glucose levels and ultimately leading to diabetes. The insulin-insulin receptor-insulin receptor substrate-1-PI3K pathway is strongly associated with insulin resistance. The 10th edition of the Global Diabetes Map reveals that approximately 537 million adults (20–79 years old) worldwide will be afflicted with diabetes in 2021, accounting for ~10.5% of the global population within this age range, and this number is projected to escalate to 643 million in 2030.^417^ Insulin resistance, type 2 diabetes, and its complications are considered chronic inflammatory diseases, and NF-κB signaling is a key pathway linking inflammation and metabolism.^182,418,419^

TANK-binding kinase 1 (TBK1), an atypical inhibitor of the IKK family, plays an important role in the regulation of inflammatory cytokine production, innate immunity, metabolism, and autophagy.^420–423^ TBK1 engages in bidirectional crosstalk in energy metabolism and inflammatory signaling.^424^ Due to the loss of mutual inhibition with the insulin receptor, the whole-genome mutation of TBK1 confers a protective effect on the metabolism of mice receiving a high-fat diet.^425–427^ The dual small molecule inhibitor of IKKε/TBK1, Amlexanox, has entered clinical trials for the treatment of obesity and type 2 diabetes.^425–427^ In contrast, it has also been demonstrated that myeloid conditional TBK1 knockout mice develop insulin resistance, nonalcoholic steatohepatitis, and experimental colitis, which may be attributed to the absence of TBK1’s role in inhibiting the NF-κB and MAPK signaling pathways played by TBK1 in M1 macrophages.^424,428^

Hyperglycemia promotes ROS production, induces oxidative stress, activates pro-inflammatory NF-κB signaling, and stimulates the immune system to release excessive inflammatory mediators and cytokines, ultimately resulting in cellular damage.^182,429^ It has been discovered that exposure to high-efficiency particulate arrestance (HEPA)-filtered air or airborne fine particulate matter (PM2.5) with a concentration of ≤2.5 μm for 9 days can lead to downregulation of IκBα levels in the aorta of mice.^430^ Furthermore, after 30 days of exposure, skeletal muscle insulin-stimulated endothelial nitric oxide synthase phosphorylation is inhibited, and adipose tissue inflammation and insulin resistance are increased.^430^ Oxidative stress and inflammation have also been shown to be major pathophysiologic changes in diabetes-induced cognitive impairment, diabetic nephropathy, diabetes-associated colitis, and dysbiosis of the colonic microbiota, among other complications.^431–434^

Diabetic nephropathy is one of the most important comorbidities in patients with diabetes mellitus, and its pathogenesis involves immunoinflammatory factors, abnormal glucose metabolism, altered renal hemodynamics, and oxidative stress. The high glucose environment induces RANK expression in glomerular podocytes, which mediates the development of diabetic nephropathy by promoting the production of ROS, TNF-α, Gal-3, and IL-1β.^432^ Kidney risk inflammatory signature (KRIS) consisting of 17 systemic proteins enriched in TNFR superfamily members has been identified to be associated with a 10-year risk of end-stage renal disease from both three independent cohorts of patients with type 1 and type 2 diabetes mellitus.^435^ Metabolic insufficiency due to mitochondrial damage is also a possible mechanism of nephritis. cGAS-STING-NF-κB pathway promotes cytoplasmic translocation of mitochondrial DNA and induces expression of pro-inflammatory factors in renal tubular epithelial cells.^436^

Hyperglycemic state in diabetic patients leads to abnormal bone metabolism, causing peri-implantitis and alveolar bone defects in the implant area, and activation of NF-κB signaling as a mechanism to maintain a long-term inflammatory state.^437^ Inhibition of NF-κB signaling activation in stem cells indirectly modulates macrophage polarization toward M1, which can restore immunoregulatory capacity and reduce local inflammation.^438^

The evolution of diabetic cardiomyopathy to myocarditis involves strong activation of NF-κB signaling, which upregulates and secretes cytokines, chemokines, and adhesion molecules expression and secretion.^439^ Downregulation of cardiac-specific Jund proto-oncogene subunit (JunD) (a member of the activator protein-1 family of transcription factors) expression and upregulation of the NF-κB inflammatory pathway are observed in the myocardium of diabetic mice induced by hyperglycemia and represent defects in myocardial antioxidant and inflammatory capacity.^440^ The level of Salusin-β, a biologically active peptide associated with the inflammatory response of vascular endothelial cells, is elevated in patients with diabetes mellitus. Investigators found that knockdown of Salusin-β effectively inhibited oxidative stress, NF-κB activation, and upregulation of inflammatory responses via NADPH oxidase 2 (NOX2)/ROS/NF-κB signaling in a rat model of diabetic cardiomyopathy, whereas the NF-κB inhibitor Bay 11-7082 attenuated inflammatory responses only.^441^

Pancreatic β-cells activate NIK in response to IL-1β and IFN-γ for non-canonical NF-κB signaling.^442^ Interestingly, the genetic silencing of NIK, a key molecule in the non-canonical pathway, does not alter the incidence of diabetes and inflammatory response in the mouse model.^443^ Studies on NF-κB signaling in β-cells have mainly focused on the canonical pathway, and further studies on the role of non-canonical signaling are needed.

#### Obesity

Over 30% of the global population is classified as overweight or obese, with the etiology of obesity being multifactorial and capable of manifesting at any stage of human development.^444^ Obesity and metabolism-related diseases such as hyperglycemia, dyslipidemia, and hypertension are categorized as “metabolic syndrome”. Activation of pro-inflammatory signals such as NF-κB, JNK, and inflammasome by overnutrition explains why obesity is often accompanied by a state of chronic low-grade inflammation and insulin resistance.^445^ Excess body fat is initially stored in subcutaneous adipose tissue, and when the threshold of adipose tissue capacity is surpassed, the fat cells undergo a series of changes, including increased inflammation, cellular hypertrophy, and insulin resistance.^446^

While metabolically activated adipose tissue macrophages have been associated with deleterious effects such as insulin resistance and steatosis, they have also been shown to facilitate lysosomal extravasation for the clearance of dead adipocytes.^447^ The high-fat-induced metabolic disturbances and inflammatory activation are not temporary, but rather a continuous state. The activation of adipose tissue macrophages was not completely eliminated in mice that stopped receiving a high-fat diet, even after 8 weeks of weight loss.^448^ Single-cell sorting in mice and humans has identified triggering receptor expressed on myeloid cells-2 (TREM2) signaling as a major pathway for adipose tissue macrophage resistance to lipid imbalance and a promising therapeutic target for metabolic diseases.^449,450^ Downregulation of zinc finger protein 423, a transcriptional corepressor of NF-κB, activates fibroinflammatory progenitor cells around the vasculature of mouse white adipose tissue and triggers a series of inflammatory signaling cascades in response to a high-fat diet.^451^ High reactive oxygen species production is detected in the hair follicle stem cells of young rats fed a high-fat diet and induces the activation of lipid droplets and NF-κB signaling via IL-1R signaling, inhibiting hair follicle regeneration and accelerating hair loss.^452^ The increased incidence of postmenopausal estrogen receptor (ER)+ breast cancer has been found to be associated with obesity, and estrone induced by ERα through activation of NF-κB signaling not only promotes the growth of breast cancer but also possesses pro-inflammatory effects.^453^ Hepatic TBK1 activity decreases in the fasting state, whereas in response to the inflammatory milieu of obesity, TBK1 activity increases and promotes fatty acid re-esterification.^454^ High-fat diet initiates cellular inflammatory responses, and inflammation also acts as an aggravating factor for obesity. TBK1 knockdown and NOD-like receptors family pyrin domain containing 12 (NLRP12, an inhibitory immune receptor) attenuate inflammasome activation and obesity by inhibiting NF-κB signaling.^424,455^ It has also been found that circadian clock impairment in adipose tissue due to NF-κB signaling may be a cause of obesity and its complications.^456^

### Nervous system diseases

#### Parkinson’s disease

Parkinson’s disease (PD) is one of the most common neurodegenerative disorders, characterized by the progressive loss of dopaminergic neurons in the substantia nigra pars compacta (SNpc), leading to motor symptoms such as rigidity, bradykinesia, and resting tremor. The key mechanisms for PD include misfolded α-synuclein aggregation, neuroinflammation, and mitochondrial dysfunction in synaptic terminals.^457,458^ As a master regulator of various cellular functions, NF-κB has been shown to involve in the above pathophysiological process. Toll-like receptors (TLRs) are a member of the pattern recognition receptor (PRR) family, which are important in the recognition of pathogens.^458^ Dutta et al. reported that α-synuclein spreading depends on the TLR2/MyD88/NF-κB pathway and can be alleviated by nasal delivery of wtTIDM and wtNBD peptides.^459^ In addition, NF-κB activation in astrocytes and microglia modulates inflammatory responses and leads to the production of pro-inflammatory molecules such as inducible nitric oxide synthase (iNOS) and tumor necrosis factor (TNF-α).^458,460^ Researchers also revealed that increased translocation of NF-κB in dopaminergic neurons can activate genes encoding pro-inflammatory mediators, which initiate the generation of reactive oxygen species (ROS) through the auto-oxidation of dopamine.^461,462^ Furthermore, microRNAs such as miR-124 can upregulate NF-κB expression, promoting pro-inflammatory levels causing neurodegeneration in PD.^463,464^ Targeting the NF-κB signaling offers a new opportunity as a therapeutic approach for PD.

#### Brain injury

Inflammation in the central nervous system (CNS) is a well-recognized feature of various acute neurological injuries.^465^ Following brain injury, the release of factors from damaged cells or cell debris may trigger an inflammatory response. Injury and ischemia can activate TLRs, which subsequently activate pro-inflammatory transcription factors such as NF-κB, leading to the upregulation of numerous immune mediators^466^ (Fig. 4i).

Inhibiting excessive inflammation after brain injury by suppressing NF-κB activation can be beneficial in protecting neuronal cells. Research has shown that geniposide exerts neuroprotective effects against traumatic brain injury (TBI) by inhibiting phosphorylation of p38 and p65 in the NF-κB pathway.^467^ Other studies have also demonstrated the preventive effect of tannic acid on brain injury in rats by modulating pathways involving Nrf2, NF-κB, and apoptosis.^468^

#### Spinal cord injury

The inflammatory response plays a significant role in the pathophysiology of acute and chronic spinal cord injury (SCI), contributing to secondary injury. The NF-κB family of transcription factors is crucial for the activation of genes involved in cell inflammation, proliferation, and cell death responses.^469^ It has been implicated as a critical determinant of cell death and central nervous system diseases. NF-κB activates the transcription of genes encoding cytokines, COX-2, CAMs, and iNOS.^470^ Inhibitors of NF-κB have potential therapeutic applications in the treatment of SCI.

Valproic acid (VPA) has been shown to increase the acetylation of the STAT1/NF-κB pathway. This promotes the transition of microglia from an M1 pro-inflammatory phenotype to an M2 anti-inflammatory phenotype, inhibiting microglial activation. Consequently, VPA reduces SCI-induced inflammatory factors and alleviates the central inflammatory response mediated by microglia after spinal cord injury.^471^Researchers have also discovered that upregulation of Sterile alpha and Toll/interleukin 1 receptor motif-containing protein 1 (SARM1)-mediated NF-κB signaling in neurons and astrocytes promotes early neuroinflammation in SCI. Therefore, targeting SARM1 with therapeutic drugs offers a promising approach to preserving neuronal function following spinal cord injury.^472^

### Corona virus disease 2019

Hyperactivation of the NF-κB pathway has been implicated in the pathogenesis of COVID-19, especially the severe phenotype.^473^ The activation of NF-κB by severe acute respiratory syndrome coronavirus 2 (SARS-CoV-2) leads to the production of pro-inflammatory cytokines, such as interleukin-6 (IL-6) and tumor necrosis factor-alpha (TNF-α).^474^ While these cytokines play a critical role in fighting off viral infections, cytokine storm can occur in severe cases of COVID-19. This overwhelming inflammatory response is associated with tissue damage and contributes to the development of acute respiratory distress syndrome (ARDS) and other complications.

During the initial stages of SARS-CoV-2 infection, NF-κB activation is critical in initiating the inflammatory response. Studies have highlighted that upon viral entry, SARS-CoV-2 engages with host ACE2 receptors and TMPRSS2 proteases, leading to viral entry and subsequent activation of NF-κB pathways and triggering of inflammatory cascades.^475,476^ The involvement of NF-κB in the outbreak stage of COVID-19 leads to an exaggerated inflammatory response characterized by the release of pro-inflammatory cytokines. This cascade of events contributes to the cytokine storm observed in severe cases, leading to tissue damage and worsening clinical outcomes.^476,477^ The role of NF-κB in the long-lasting stage of COVID-19 infections involves persistent inflammatory responses, potential tissue damage, and complications leading to prolonged symptoms or post-acute sequelae of SARS-CoV-2 infection (PASC). Research indicated that NF-κB activation can perpetuate the inflammatory environment even after the initial infection subsides. Carfì et al. observed persistent inflammatory markers in patients recovering from acute COVID-19, suggesting a role for NF-κB in sustaining inflammation and potentially contributing to the development of PASC.^478^ These studies suggested that NF-κB activation during this stage may contribute to chronic inflammation and immune dysregulation, influencing the extended duration and severity of symptoms.

Although NF-κB activation induces the expression of antiviral proteins to help control viral replication, SARS-CoV-2 has evolved mechanisms to evade the host immune response, including inhibiting some aspects of NF-κB signaling.^479^ The complex interaction between the virus and NF-κB signaling in COVID-19 is still being actively investigated. Targeting NF-κB signaling pathways may hold potential for therapeutic interventions, but further studies are needed to determine the efficacy and safety of such approaches.

## Strategies for targeting NF-κB signaling in human diseases

### IKK inhibitors

#### Aspirin

**Fig. 5:**
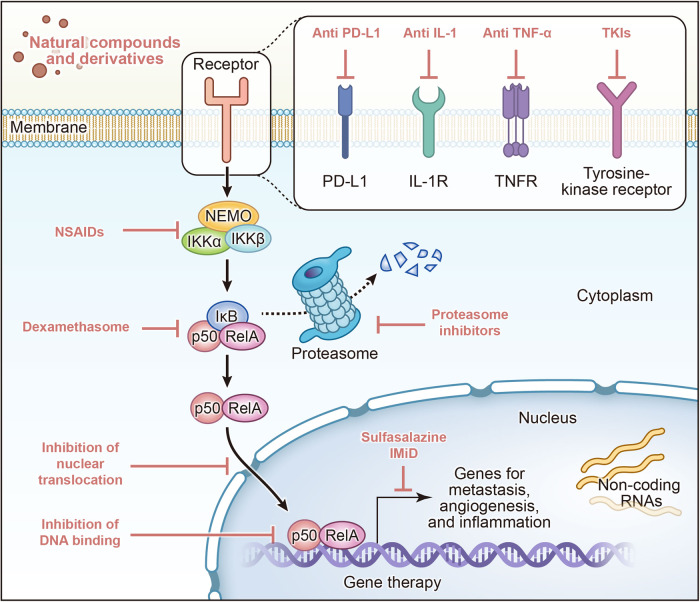
Strategies for targeting NF-κB signaling in human diseases. Inhibitors of NF-κB are widely used in various clinical settings for the treatment of tumors, diabetes, and other conditions. These drugs inhibit the NF-κB pathway through different mechanisms. NSAIDs selectively inhibit IκB to suppress the activation of NF-κB. Dexamethasone inhibits NF-κB by directly coupling with the RelA subunit to block its functional activity. IMiD drugs such as thalidomide suppress the transcription function downstream of NF-κB to exert their effects. Monoclonal antibodies, including anti-PD-L1, anti IL-1, and anti TNF-α block the binding of ligands and respective receptors to inhibit their biological effects. Proteasome inhibitors include Bortezomib, Carfilzomib, Ixazomib, and Lactacystin act by halting protein degradation that ultimately results in apoptosis and cell death. Tacrolimus inhibits the protein phosphatase activity of calcineurin, preventing the nuclear translocation of NFAT and subsequently suppressing the activation of T cells. IκBα super-repressor inhibits the translocation of NF-κB into the nucleus. Tyrosine kinase inhibitors inhibits the intracellular phosphorylation of tyrosine kinase to block tumor cell growth. Natural compounds and derivatives such as resveratrol, quercetin, and isothiocyanates inhibits NF-κB through diverse mechanisms and exerts antitumor effects. Strategies targeting non-coding RNAs are also in development, such as anti-miR oligonucleotides, which can be used to inhibit miRNAs that promote the NF-κB signaling pathway. NF-κB-activated gene expression is a novel gene therapy to treat cancer by utilizing overactivation of NF-κB in cancer cells. IL-1 interleukin 1, IMiD immunomodulatory drugs, NFAT nuclear factor of activated T cells, NSAIDs nonsteroidal anti-inflammatory drugs, PD-L1 programmed death-ligand 1, TNF-α tumor necrosis factor α

Aspirin, a nonsteroidal anti-inflammatory drug (NSAID), is well-known for its action in inhibiting cyclooxygenase. Elizabeth Kopp et al. first discovered in 1994 that anti-inflammatory drugs such as sodium salicylate and aspirin can inhibit the degradation of IκBα and prevent NF-κB-dependent transcription, which further confirms the important role of NF-κB signaling in inflammation and infection.^480^ Research suggests that the anti-inflammatory properties of aspirin are partially attributed to its specific inhibition of IKKβ, thereby preventing the activation of NF-κB and genes associated with inflammatory responses^481^ (Fig. 5).

Furthermore, substantial evidence indicates that aspirin and related NSAIDs possess potential antitumor activity and cancer-preventive effects, leading to increased interest in using aspirin for cancer treatment. Mechanistically, aspirin reduces the migration, invasion, and metastasis of osteosarcoma cells through the modulation of the NF-κB pathway.^482^ Hydrogen sulfide-releasing aspirin (HS-ASA), a novel derivative of aspirin, inhibits the growth of MDA-MB-231 breast cancer cells by downregulating the NF-κB pathway, inducing cell cycle arrest, and promoting apoptosis. HS-ASA also affects thioredoxin reductase activity and increases reactive oxygen species levels.^483^ Additionally, aspirin induces apoptosis in human colorectal cancer cells by inhibiting NF-κB activity, making it a potential therapeutic agent for colon cancer^484^ (Table 2).

**Table 2:** Summary of mechanisms and main references of strategies targeting NF-κB signaling for the treatment of human diseases

Name	Condition	Mechanism	Reference
**A. IKK inhibitors**			
Aspirin	Inflammation, infection, cancer	Inhibition of IKKβ	^480–484^
Sodium Salicylate	Inflammation, leukemia	Inhibition of IKKβ	^485–488^
Sulfasalazine	Inflammation, infection, cancer, cardiovascular disease	Inhibition of TLR4, MyD88, and p65	^489–493^
Dexamethasone	Inflammation, infection, lymphoma, leukemia	Inhibition of RelA	^494–498^
Thalidomide	Infection, cancer, leukemia	Inhibition of NF-κB activation	^499–503^
Lenalidomide	Myeloma, lymphoma	Inhibition of NF-κB and TNF-α activation	^504–507^
Pomalidomide	Myeloma, lymphoma	Inhibition of NF-κB activation and suppression of COX-2 gene transcription	^508–511^
Pyridine derivative compound A	Inflammation, cancer, diabetes	Inhibition of NF-κB activation	^512–514^
BAY 11-7821	Inflammation, infection, cancer	Reduce the expression of NF-κB and adhesion molecules	^515^
Bardoxolone methyl	Inflammation, cancer, cardiovascular diseases, neurodegenerative diseases	Inhibition of NF-κB activation	^516^
Andrographolide	Inflammation, infection, cancer	Modify the cysteine residue of p50	^517^
**B. Monoclonal antibody**			
Anti-PD-1/PD-L1	Cancer	Block the PD-1/PD-L1 interaction	^533^
Anti IL-1	Rheumatoid arthritis, auto-inflammatory diseases, CAPS, AOSD, cancer	Inhibit activation of NF-κB signaling	^540^
Anti-β2-microglobulin monoclonal antibodies	Multiple myeloma	Reduce NF-κB activity	^541,542^
**C. Proteasome inhibitors**			
Bortezomib	Multiple myeloma, diffuse large B cell lymphoma	Target the unfolded protein response (UPR) pathway to inhibit IκBα degradation	^548,549^
Carfilzomib	Multiple myeloma		^550,553^
Ixazomib	Multiple myeloma		^554^
Marizomib	Multiple myeloma		^555–557^
**D. Inhibition of nuclear translocation**			
Tacrolimus	Prevent organ rejection after transplantation, atopic dermatitis, IBD	Block NFAT nuclear translocation	^562,565^
IκBα super-repressor	Alcohol-associated liver injury, sepsis-associated organ damage, kidney ischemia-reperfusion injury, and amyotrophic lateral sclerosis (ALS)	Inhibit NF-κB by preventing nuclear translocation	^566–568^
**E. Inhibition of DNA binding**			
Glucocorticoids	Allergic reactions, autoimmune disorders (rheumatoid arthritis, systemic lupus erythematosus, multiple sclerosis, etc.), asthma, IBD	Inhibit NF-κB DNA binding	^573,574^
PPAR agonists	Diabetes, inflammation, neurodegenerative diseases, and cancer	Inhibit NF-κB DNA binding	^581,582^
**F. Tyrosine kinase inhibitors**	Cancer	Inhibition or induction of NF-κB signaling	^584–587^
**G. Non-coding RNAs**	Cancer	Engage in the aberrant regulation of NF-κB signaling	^589,590^
**I. CAR-T**	Cancer	Genetic engineering of a patient’s own T cells to express a CAR that can recognize and bind to specific tumor-associated antigens	^598,600–602^

#### Sodium salicylate

Sodium salicylate, similar to aspirin, is an NSAID. It has been discovered that sodium salicylate inhibits the activation of the transcription factor NF-κB.^485^ This finding opens up opportunities for exploring new applications of sodium salicylate.

In the context of anti-inflammatory therapy, both acetylsalicylic acid (aspirin) and its metabolite sodium salicylate have been found to protect against excitotoxicity caused by glutamate in neuronal cultures and hippocampal slices. This neuroprotective effect is achieved through the inhibition of NF-κB.^486^ Sodium salicylate is known as a COX-2 inhibitor and has been shown to improve insulin secretion defects in diabetic patients. It has been reported that this drug can prevent the dissociation of NF-κB from the NF-κB/IκB complex, thereby preventing the translocation of NF-κB from the cytoplasm to the nucleus and inhibiting the transcription of COX-2.^487^ Furthermore, sodium salicylate induces a shift from a proliferative to an apoptotic phenotype in human leukemia cells by inhibiting the NF-κB response and restoring TNF-induced apoptosis.^488^

#### Sulfasalazine

Sulfasalazine is primarily used as a sulfonamide antibiotic. When partially absorbed, it undergoes breakdown by intestinal microbiota into 5-aminosalicylic acid (5-ASA) and sulfapyridine (SP). 5-ASA complexes with the connective tissue of the intestinal wall, exert antimicrobial, anti-inflammatory, and immunosuppressive effects. It inhibits the synthesis of prostaglandins and other inflammatory mediators like leukotrienes.^489^ Sulfasalazine (SAS) is a known NF-κB inhibitor that can inhibit the expression of TLR4, MyD88, and NF-κB p65 proteins induced by trinitro-benzene-sulfonic acid (TNBS).^490^

Neointimal hyperplasia (NH) induced by arterial injury is a major cause of arterial stenosis. Sulfasalazine prevents post-injury vascular stenosis by inhibiting the proliferation and migration of vascular smooth muscle cells through the NF-κB/mTOR pathway.^491^ Additionally, sulfasalazine plays a significant role in cancer treatment. It can promote apoptosis in U251 glioblastoma cells by inhibiting NF-κB signaling.^492^ The IL-1β-NFKB/CREB-Wnt signaling pathway has also been identified as a novel mechanism promoting breast cancer stem cell (CSC) colonization in the bone microenvironment. Targeting this pathway with drugs like sulfasalazine can prevent in vivo bone metastasis and colony formation of breast CSCs in vitro.^493^

#### Dexamethasone

Dexamethasone, a glucocorticoid, exhibits various pharmacological effects, including anti-inflammatory, anti-endotoxin, immunosuppressive, anti-shock, and stress response enhancement properties.^494^ The inhibition of NF-κB activation is one of the possible mechanisms by which dexamethasone exerts its therapeutic effects. There are two proposed mechanisms for this inhibition: Activated glucocorticoid receptors directly interact with the RelA subunit of NF-κB in the cell nucleus, leading to the inhibition of its function. Activated glucocorticoid receptors enhance the transcription of IκB and increase its levels. This, in turn, prevents the nuclear translocation of NF-κB and its binding to DNA.^495^ These mechanisms provide a basis for the therapeutic application of dexamethasone in diseases involving NF-κB dysregulation.

Studies have demonstrated that dexamethasone can alleviate acute pancreatitis and liver injury by inhibiting the NF-κB pathway.^496^ In the context of arthritis, dexamethasone can treat the condition and alleviate joint swelling symptoms by inhibiting the expression of the p65 protein in the NF-κB pathway.^497^ Additionally, in oral lichen planus (OLP), where the TLR4-NF-κB-p65 axis plays a crucial role, dexamethasone effectively protects against epidermal cell damage by downregulating TLR4 expression and negatively regulating the NF-κB signaling pathway in keratinocytes.^498^ These findings highlight the therapeutic potential of dexamethasone in NF-κB-mediated diseases and conditions. Its ability to inhibit NF-κB activation contributes to its anti-inflammatory effects and provides a rationale for its use in various inflammatory disorders.

#### Thalidomide

Thalidomide, initially developed as an anti-leprosy medication, has been found to have various pharmacological effects. Its mechanism of action involves immunosuppression, immune modulation, and inhibition of neutrophil chemotaxis. Multiple studies have indicated that thalidomide achieves these effects by inhibiting NF-κB activation.^499,500^

In terms of inflammatory therapy, thalidomide has demonstrated the ability to improve rosacea-like skin inflammation by inhibiting NF-κB activation in keratinocytes.^501^ Additionally, thalidomide may have therapeutic potential in tumor treatment. It can inhibit tumor necrosis factor α-induced ICAM-1 expression by suppressing the NF-κB-binding ICAM-1 promoter, leading to the inhibition of proliferation in lung cancer cells.^502^ Notably, the mechanism behind thalidomide-induced limb malformation is also related to NF-κB. Research has revealed that species-specific changes in the redox microenvironment, triggered by free radical generation from thalidomide, lead to the suppression of NF-κB-mediated gene expression, which is accountable for phocomelia (a congenital malformation of the limbs resembling a seal’s flippers).^503^

#### Lenalidomide

Lenalidomide, an analog of thalidomide, is an antitumor drug with various therapeutic effects, including antitumor activity, immune modulation, and anti-angiogenesis properties.^504^ Like thalidomide, lenalidomide also possesses the ability to inhibit NF-κB. However, it exhibits significantly higher potency in inhibiting TNF-α in vitro than thalidomide, with a 50,000-fold difference.^505^

Lenalidomide received FDA approval in 2003 for the treatment of relapsed or refractory multiple myeloma. It impairs the NF-κB signaling pathway in bone cells, resulting in the suppression of osteoclast-specific gene expression. This provides therapeutic effects against bone resorption and makes lenalidomide a valuable treatment option for osteolytic diseases such as multiple myeloma.^506^ In addition, lenalidomide has shown promise in the treatment of DLBCL. Its antitumor effect in DLBCL cells is associated with the downregulation of IRF4 and subsequent inhibition of B-cell receptor-dependent NF-κB activity.^507^

#### Pomalidomide

Pomalidomide, a next-generation immunomodulatory drug (IMiD), is primarily used in the treatment of relapsed/refractory multiple myeloma. It offers improved efficacy and toxicity characteristics compared to its sister compounds, lenalidomide and thalidomide.^508^ In MM cells, which heavily rely on various transcription factors, pomalidomide has been observed to inhibit NF-κB transcription and suppress COX-2 gene transcription activity, contributing to its therapeutic effects.^509^

Research is also being conducted to explore the potential use of pomalidomide in other diseases. For instance, it has shown the ability to enhance chemotherapy sensitivity in pancreatic cancer by inhibiting NF-κB activation induced by chemotherapy.^510^ Additionally, pomalidomide has demonstrated the capacity to suppress NF-κB levels and significantly reduce cortical neuron apoptosis by regulating Bax, cytochrome c, and poly (ADP-ribose) polymerase. This makes it a potential therapeutic agent for brain injury and related disorders.^510,511^

#### Pyridine derivative compound A

Pyridine and its derivatives have shown diverse biological activities and have been utilized in the synthesis of various drugs. Many of these drugs exhibit NF-κB inhibitory activity, which contributes to their pharmacological effects. Two examples of such drugs are imatinib and moxifloxacin.^512^

Imatinib is a well-known drug used for the treatment of diabetes and chronic myeloid leukemia (CML). During imatinib therapy, several transcription factors, including NF-κB, are activated in response to physiological and pathological changes. Studies have observed that the release of IL-6 and IL-8, as well as the activation of NF-κB and AP-1, were significantly reduced in lymphomonocytes of imatinib-treated patients. These findings suggest that the downregulation of these factors can potentially serve as favorable prognostic indicators for improved outcomes in patients receiving imatinib therapy.^513^ Moxifloxacin, on the other hand, is an antibiotic that exhibits anti-inflammatory effects. It achieves this by inhibiting the activation of NF-κB and mitogen-activated protein kinase in monocytes. Additionally, moxifloxacin suppresses the synthesis of pro-inflammatory cytokines, further contributing to its anti-inflammatory properties.^514^

#### New type inhibitors

From the first generation to the second generation of IKKβ inhibitors, there have been significant improvements in both the selectivity and potency of the drugs. Many IKKβ inhibitors have entered the preclinical research stage. BAY 11-7821 is an inhibitor of IκBα phosphorylation and NF-κB activation. It selectively and irreversibly inhibits TNF-α-induced IκB-α phosphorylation and reduces the expression of NF-κB and adhesion molecules. It exhibits high selectivity, primarily targeting the IKKβ subunit and does not significantly affect other related protein kinases.^515^ Bardoxolone methyl is a synthetic triterpenoid compound that has demonstrated inhibitory effects on NF-κB in various areas such as kidney disease and chronic obstructive pulmonary disease. Besides inhibiting NF-κB, Bardoxolone methyl also affects signaling pathways such as Nrf2 and PPAR-γ, giving it a broad therapeutic potential.^516^ Andrographolide is an NF-κB inhibitor derived from the Chinese medicinal herb *Andrographis paniculata*. It inhibits NF-κB activation by covalently modifying the cysteine residue of p50 in endothelial cells, without affecting IκBα degradation or p50/p65 nuclear translocation.^517^

### Monoclonal antibody

Monoclonal antibodies mainly target NF-κB signaling through the inhibition of ligand-receptor interaction. Typical agents include anti-PD-1/PD-L1 and anti-IL-1. Besides, some novel monoclonal antibodies specifically targeting NF-κB signaling are under development.

#### Anti-PD-1/PD-L1

Programmed death-Ligand 1 (PD-L1) expression is upregulated in tumor cells or antigen-presenting cells, and evades immune surveillance after binding to programmed death 1 (PD-1) on the surface of tumor-infiltrating immune cells.^518,519^ RelB promotes prostate cancer immune evasion by expanding PD-L1/PD-1-mediated immune checkpoints to suppress T-cell immunity.^520^ TNF-α inhibits ubiquitinated degradation of PD-L1 via p65-induced COP9 signalosome 5 (CSN5), leading to immune escape.^521^ Immunotherapy resistance is an area of concern. TRAF2-deficient multiple myeloma cells enhance immunomodulatory drug resistance through activation of non-canonical NF-κB signaling and ERK signaling.^522^ Low MHC-I expression may lead to resistance to immune checkpoint inhibitors by inhibiting the IFN-γ signaling pathway, whereas guanine nucleotide-binding protein subunit gamma 4 (GNG4) maintains MHC-I expression through the NF-κB signaling.^523^ Inhibitors of the deubiquitinating enzyme ubiquitin-specific proteases (USP) 8 activate NF-κB signaling to trigger innate immune responses and MHC-I expression, thereby remodeling the inflammatory tumor microenvironment and enhancing the antitumor efficacy of anti-PD-1/PD-L1 therapies.^524^ CD11b agonists activate TAM and resist immunosuppression by degrading p65 and activating STING/STAT1 signaling.^525^

Anti-PD-1/PD-L1 antibodies are representative immune checkpoint inhibitors widely used in cancer therapies.^526,527^ These agents act by blocking the PD-1/PD-L1 interaction, thereby releasing the brakes on the immune system and combating cancer immune evasion. Currently, FDA-approved anti-PD-1/PD-L1 antibodies include pembrolizumab, atezolizumab, avelumab, durvalumab, and nivolumab.^528–531^ However, it is noteworthy that not all patients respond equally to these immunotherapies, and further studies to identify predictors of response and pathways strengthening the efficacy are needed to optimize their clinical use.^518,532^ Intriguingly, the NF-κB pathways have recently been raised as promising molecular targets to enhance the antitumor activity of checkpoint inhibitors. It has been shown that activation of the alternative NF-κB pathway combined with anti-PD-1 treatment can yield a complete and durable antitumor response in xenograft of melanoma and colorectal tumors.^533^ The upregulation of alternative NF-κB pathway in tumor-infiltrating DCs can be induced by anti-PD-1 treatment, leading to subsequent secretion of IL-12, which enhanced CD8 + T cell antitumor activity. Based on these findings, clinical trials investigating the combination of PD-1 inhibitors and agonistic CD40 mAbs/SMAC mimetics (which activate the alternative NF-κB signaling) for cancer treatment are currently ongoing (NCT03123783, NCT02376699, and NCT03270176).^534^

#### Anti IL-1

Anti-IL-1 inhibits the activity of interleukin-1 (IL-1), which plays a crucial role in inflammation via activation of canonical NF-κB signaling and is associated with various autoimmune and inflammatory conditions such as rheumatoid arthritis, gout, and certain skin diseases. IL-1 inhibitors, including canakinumab, rilonacept, and anakinra, have been approved for the treatment of rheumatoid arthritis,^535^ auto-inflammatory diseases,^536^ cryopyrin-associated periodic syndromes (CAPS),^537,538^ adult-onset Still’s disease (AOSD).^539^ Their applications in cancer therapy have also been under investigation. However, due to the dual role of IL-1 in tumor development, direct inhibition failed to yield satisfactory efficacy, posing challenges to the development of antitumor drugs targeting IL-1. The combination of IL-1 inhibitor with a PD-1 inhibitor in a recent clinical trial has shown clinically meaningful delays in the deterioration of symptoms for the treatment of lung cancer, although did not prolong PFS or OS (NCT03631199).^540^

#### Anti-β2-microglobulin monoclonal antibodies

Specific monoclonal antibodies that target both tumor cells and the tumor microenvironment are currently under development. One promising antibody-based novel agent in multiple myeloma is anti-β2-microglobulin monoclonal antibodies, which have shown remarkable antitumor activity on myeloma both in vitro and in xenograft with low toxicity.^541,542^ Studies have demonstrated that combining bortezomib with anti-β2-microglobulin monoclonal antibodies can significantly reduce NF-κB activity, induce tumor cell apoptosis, and overcome bortezomib resistance. This combination therapy also inhibits bortezomib-induced autophagy mediated by the interaction of p65 with the beclin 1 promoter.^543^ The enhanced effect of the combination therapy holds promise as it may allow for lower doses of either substance, reducing toxicity while enhancing efficacy. These studies highlight the potential of antibody-based therapies in multiple myeloma treatment and their ability to modulate the NF-κB signaling pathway.

### Proteasome inhibitors

Proteasome inhibitors mainly target the unfolded protein response (UPR) pathway to inhibit IκBα degradation and thus suppress NF-κB. Suppression of proteasome leads to the accumulation of misfolded proteins and subsequent endoplasmic reticulum stress in cancer cells, inducing cell cycle arrest and apoptosis.^544^ FDA-approved proteasome inhibitors include bortezomib, carfilzomib, and ixazomib, which have been mainly used for the treatment of multiple myeloma, diffuse large B cell lymphoma (DLBCL), and other solid tumors.^545^

#### Bortezomib

Bortezomib is a first-in-class selective and reversible proteasome inhibitor, which binds to and inhibits the activity of the 26 S proteasome, a cellular complex responsible for degrading and recycling proteins.^546,547^ By blocking the proteasome’s function, bortezomib was initially thought to inhibit IκB degradation, which is necessary for NF-κB activation. However, it has been later confirmed that bortezomib can also induce activation of canonical NF-κB signaling in multiple myeloma in vitro.^548^ Further investigations indicated that this contradictory effect can be prevented by combination therapies such as the use of calpain inhibitors.^549^

#### Carfilzomib

Carfilzomib is a second-generation proteasome inhibitor targeting 20 S proteasome. Unlike bortezomib, carfilzomib irreversibly binds to the active site of the proteasome with higher selectivity, leading to a prolonged inhibition of its activity.^550^ This mechanism allows carfilzomib to exert a more sustained and profound effect on proteasome function. Additionally, carfilzomib has almost no off-target activity outside of proteasome and has been confirmed to inhibit NF-κB signaling in recent studies. Carfilzomib has demonstrated significant efficacy in patients with relapsed or refractory multiple myeloma in clinical trials, either as a single agent or in combination with other anticancer therapies.^551–553^

#### Ixazomib

Ixazomib is an oral second-generation proteasome inhibitor, acting by selectively and reversibly inhibiting the 20 S proteasome. Its oral formulation allows for easier administration and provides greater accessibility for long-term treatment. Ixazomib can effectively inhibit both activation pathways of NF-κB in MM stromal cells.^554^ Ixazomib is often used in combination with other anticancer drugs and has shown significant efficacy in clinical trials for the treatment of multiple myeloma. It provides an additional option for patients, expanding the range of treatment strategies available to improve outcomes and quality of life for those living with multiple myeloma.^545^

#### Marizomib

Marizomib is a novel, irreversible proteasome inhibitor developed for the treatment of relapsed or relapsed and refractory multiple myeloma. It inhibits the 3 proteolytic activities of the 20 S proteasome with specificity distinct from bortezomib and carfilzomib. Marizomib also inhibits NF-κB signaling through inhibition of IκBα degradation. Preclinical studies and early-phase clinical trials (NCT00461045, NCT00629473) have shown promising results for marizomib in terms of its anticancer activity in multiple myeloma and tolerability.^555–557^ Its use in other solid tumors such as glioblastoma and breast cancer are under preclinical investigations.^558,559^ Further clinical trials are still ongoing to evaluate its effectiveness as a single agent or in combination with other therapies for treating cancer.

### Inhibition of nuclear translocation

#### Tacrolimus

Tacrolimus (FK506) is an immunosuppressive agent that is primarily used to prevent organ rejection after transplantation.^560,561^ It inhibits the activity of calcineurin, an enzyme that plays a crucial role in the activation of the nuclear factor of activated T cells (NFAT). By blocking NFAT nuclear translocation, tacrolimus inhibits the activation of NF-κB signaling, and thus suppress the activity of various immune cells and alleviate inflammation. Apart from preventing organ rejection, tacrolimus is also prescribed for certain dermatological conditions, such as moderate to severe atopic dermatitis (eczema) when other treatments have proven ineffective.^562,563^ Increasing evidence has supported tacrolimus to be used as a second-line therapeutic agent for ulcerative colitis and Crohn’s disease.^560,562,564,565^ Currently, tacrolimus demonstrates a relatively pronounced short-term induction of remission and is gradually being utilized in the management of traditional drug-resistant or anti-TNF-resistant IBD, while evidence regarding its long-term efficacy and safety with prolonged use remains limited.

#### IκBα super-repressor

The IκBα super-repressor is a genetically engineered IκB protein without IKK phosphorylation sites, leading to a sustained inhibition of NF-κB by preventing nuclear translocation. As a result, the downstream genes regulated by NF-κB is repressed. Emerging preclinical evidence has addressed the wide use of IκBα super-repressor delivered through exosome systems in various diseases, including alcohol-associated liver injury, sepsis-associated organ damage, kidney ischemia-reperfusion injury, and amyotrophic lateral sclerosis (ALS).^566–570^ By utilizing an engineered exosome technology called “exosomes for protein loading via optically reversible protein–protein interactions (EXPLOR)”, Yim et al., engineered exosomes to load super-repressor IκB (Exo-srIκB) for the efficient intracellular transfer of protein-based therapeutics.^571^ Although the application of IκBα super-repressor is currently limited to laboratory research settings, it demonstrates high potential as a promising intervention in clinical practice in the future.

### Inhibition of DNA binding

#### Glucocorticoids

Glucocorticoids exert their effects by binding to specific receptors inside cells, known as glucocorticoid receptors (GRs). The precise effect of glucocorticoids on DNA binding and transcriptional regulation is complex and context-dependent. On one hand, glucocorticoid binding to the glucocorticoid response elements (GREs) can enhance gene transcription, leading to increased protein synthesis. This activation of gene expression can be seen with certain genes involved in anti-inflammatory responses or metabolic processes. On the other hand, Glucocorticoids can suppress DNA binding and gene expression by various mechanisms. GR activation can cause genome-wide blockade of NF-κB, inhibiting their ability to bind to DNA and suppressing the expression of pro-inflammatory genes.^572–574^ Additionally, the activated GRs can recruit co-repressors, which further inhibit the binding of other transcription factors or interfere with the assembly of the transcription initiation complex.

Due to their potent anti-inflammatory and immunosuppressive properties, glucocorticoids are commonly used for the treatment of a wide range of inflammatory conditions, including allergic reactions (skin rashes, bronchial constriction, etc.), autoimmune disorders (rheumatoid arthritis, systemic lupus erythematosus, multiple sclerosis, etc.), asthma, IBD (Crohn’s disease, ulcerative colitis, etc.).

#### PPAR agonists

PPAR (peroxisome proliferator-activated receptor) agonists activate one or more isoforms of the PPAR family. There are three main isoforms of PPARs: PPAR-α, PPAR-δ/β, and PPAR-γ, each of them has distinct tissue distribution and biological functions.^575–578^ PPAR agonists can selectively target one or more isoforms to elicit specific therapeutic effects.^579,580^

PPAR-α agonists (e.g., fenofibrate and gemfibrozil) primarily target PPAR-alpha and are used to treat dyslipidemia and reduce triglyceride levels. They promote fatty acid oxidation in the liver, leading to increased clearance of triglycerides from the blood. PPAR-δ/β agonists, such as GW501516 (also known as cardarine), can enhance fatty acid metabolism, improve insulin sensitivity, and modulate skeletal muscle function. PPAR-δ/β agonists have shown potential for treating metabolic disorders such as obesity, dyslipidemia, and type 2 diabetes. PPAR-γ agonists, including pioglitazone and rosiglitazone, are widely used for managing insulin resistance and improving glycemic control in individuals with type 2 diabetes. They can enhance insulin sensitivity, promote glucose uptake by peripheral tissues, and regulate adipocyte differentiation and lipid metabolism. Additionally, investigations are ongoing to explore other potential use of PPAR agonists in various conditions, such as inflammation, neurodegenerative diseases, and cancer. It is noteworthy that studies have suggested that certain PPAR agonist (thiazolidinedione) can inhibit NF-κB DNA binding, offering a new therapeutic strategy for lymphoblastic leukemia and IBD.^581,582^

### Tyrosine kinase inhibitors

Tyrosine kinase inhibitors (TKIs) are widely used in cancer therapy, acting by binding to the ATP-binding site of tyrosine kinases, preventing them from phosphorylating their target proteins. This disruption of kinase activity can inhibit downstream signaling pathways that promote cancer cell growth and survival. TKIs have shown efficacy in treating various types of cancer, including chronic myeloid leukemia (CML), gastrointestinal stromal tumors (GISTs), non-small cell lung cancer (NSCLC), renal cell carcinoma (RCC), and breast cancer.^583^

Numerous researches have suggested the interaction between TKIs and NF-κB activity. However, the relationship between TKIs and NF-κB can be complex and context-dependent. Some studies have reported that TKIs such as imatinib can suppress NF-κB signaling in certain types of cancer cells, leading to reduced cell survival and proliferation.^584^ On the other hand, there are also reports suggesting that some TKIs can induce NF-κB activity. The activation of NF-κB mediates resistance to TKI treatment.^585–587^ Targeting the NF-κB pathway may be a potential therapeutic approach to tackle TKI resistance.

### Non-coding RNAs

Non-coding RNAs (ncRNAs) mainly include microRNA (miRNA), long non-coding RNA (IncRNA), and circular RNA (circRNA).^588^ These ncRNAs play important roles in various cellular processes, including gene regulation and epigenetic modulation. Increasing evidence has demonstrated that ncRNAs may engage in the aberrant regulation of NF-κB signaling.^589^ RNAs can act more rapidly and diversely than proteins upon stimulation, which makes them significant in the regulation of NF-κB signaling. Anti-miRNA oligonucleotides have shown effectiveness in inhibiting NF-κB in cancer.^590^ Targeting NF-κB signaling by ncRNAs is an emerging therapeutic strategy of cancer treatment.

### Immunotherapy

Scientists have developed an inhibitor called KIC-0101, which effectively blocks the NF-κB pathway and inhibits the induction of pro-inflammatory cytokines both in vitro and in vivo. In a mouse model of rheumatoid arthritis, treatment with KIC-0101 significantly improves cartilage damage and inflammation.^591^ Additionally, researchers have discovered that Perillyl alcohol (POH) effectively ameliorates arthritis in rats by modulating the TLR4/NF-κB signaling pathway.^592^ The current research has identified several novel targets for treating osteoarthritis (OA) through the NF-κB pathway. For instance, the regulatory role of miR-214-3p in cartilage degradation in OA via the NF-κB pathway has been revealed, highlighting the potential of miR-214-3p as a therapeutic target.^593^

Current and future research directions mainly focus on the interplay between NF-κB and inflammation response as well as neural damage, aiming to identify therapeutic targets for improving disease progression and quality of life in multiple sclerosis (MS) patients. Sunny Malhotra et al. discovered that NF-κB is involved in the regulation of NLRP3 inflammasome, serving as a prognostic factor and potential therapeutic target in primary progressive multiple sclerosis.^594^ Furthermore, inhibition of poly (ADP-ribose) polymerase 1 (PARP1), an upstream regulator of NF-κB signaling, has demonstrated therapeutic potential in multiple sclerosis and its animal models.^595^

Mohamed El-Sherbiny et al. discovered that betulin demonstrates anti-inflammatory and anti-apoptotic effects in experimental ulcerative colitis through modulation of the TLR4/NF-κB/caspase signaling pathway.^596^ However, a significant limitation for its clinical application is its poor solubility in aqueous media, which calls for further research to enhance the drug’s clinical potential and expand its therapeutic applicability.

Inhibitors of NF-κB may also well improve the complications of SLE. The clinical implications of RIG-I hyperactivation caused by a novel pathogenic variant in DDX58 are explored, shedding light on its relevance to lupus nephritis and the potential involvement of the NF-κB pathway.^597^

### CAR (chimeric antigen receptor)-T cell immunotherapy

CAR-T cell immunotherapy is an innovative and promising approach in cancer treatment. Huang et al. has recently revealed the key role of NFAT and NF-κB in the dynamic co-regulation of TCR and CAR signaling responses in human T cells.^598^ Despite the remarkable clinical success of CAR-T in the treatment of hematological malignancies, it also faces challenges such as T cell exhaustion. Second-generation CAR targets are thus developed, including co-stimulatory regulators 4-1BB (CD137), to increase T cell expansion and delay apoptosis.^599^ Recent studies have reported that human CAR with 4-1BB endodomain leads to strong NF-κB activation through the recruitment of TRAF molecules, indicating the important role of TRAF-NF-κB axis in CAR-T persistence upon antigen stimulation.^600^ Disruption of TRAF2 signaling inhibited IKKα and IKKβ phosphorylation and prevented tonic CAR signaling-dependent T cell toxicity. A recent study showed that cis ligation of 4-1BB relative to the TCR-CD3 complex lead to more intense canonical and non-canonical NF-κB signaling, providing a more robust induction of cell cycle and DNA damage repair gene expression.^601^ In addition, Jakrawadee et al has developed a composite co-stimulatory domain of a B cell signaling moiety, CD79A/CD40, to synergize with other T cell signals and enhance CAR-T cell function. In the preclinical model, CD79A/CD40 incorporating CD19CAR-T cells demonstrated higher NF-κB and p38 activity compared with the CD28 or 4-1BB incorporating CD19CAR-T cells and improved antitumor efficacy.^602^ Further studies are still required to optimize the design of CARs and improve CAR-T cell function and persistence.

### Future directions

In summary, NF-κB signaling pathway plays a crucial role in multiple human diseases, as it operates at multiple levels of immune responses and contributes to the inflammatory lesions observed in these conditions. Therefore, developing therapeutic approaches targeting specific effectors implicated in these diseases is of utmost importance. However, the NF-κB signaling pathway encompasses multiple components and exerts its influence in various physiological responses, making it challenging to avoid potential side effects associated with NF-κB inhibitors. Hence, the future research focus lies in developing more specific NF-κB inhibitors for the treatment of inflammatory disorders.

Prospectively, strategies targeting NF-κB signaling require development in the following directions: (1) Developing NF-κB inhibitors that simultaneously target multiple key nodes; (2) Improving the bioavailability, safety, and stability of small molecule drugs; (3) Enhancing drug specificity to minimize interference with other cellular functions; (4) Exploring natural products and traditional Chinese medicine research.

## Conclusion

In the field of NF-κB signaling, there have been several outstanding reviews describing the components and transmission mechanisms of canonical or non-canonical NF-κB signaling.^33,83,183,189,191,217,306,603–609^ Regarding the biological functions of NF-κB signaling, the main focus has been on inflammation, immune response, and metabolism. In conclusion, the attention of these articles centers on NF-κB signaling itself. In this review, we attempt to take a broader perspective on NF-κB signaling by describing its involvement in various human diseases and summarizing the therapeutic means for targeting NF-κB signaling, which is an important characteristic that distinguishes this review from others. We hope to be able to provide reference to scholars (beginners, scientists, or clinicians) from multiple intellectual profiles in this format.

More is known about NF-κB signaling and more is unknown. The timing of activation can affect the transcriptional responses produced by NF-κB signaling in response to stimuli. Oscillatory activation of NF-κB promotes transcription of inflammatory genes, whereas persistent activation reprograms the epigenome, involving a broader range of genes.^610^ Sine oculis homeobox (SIX) family transcription factors are activated through non-canonical NF-κB signaling and then bind to the promoter regions of pro-inflammatory genes to directly inhibit RELA and RELB function, a negative feedback loop that enriches the understanding of NF-κB signaling and provides a possible therapeutic target.^611^ Components of NF-κB signaling also act independently, a prime example being the finding that IKK controls Rel-deficient thymocytes from RIPK1-dependent cell death independently of NF-κB activation.^612^

The involvement of NF-κB signaling in inflammation and the immune response is re-emphasized in this review, particularly in the context of immunotherapy as a “revolution” for tumors and other diseases. Indeed, NF-κB signaling has demonstrated initial potential in a variety of diseases thought to be difficult to overcome, extending beyond those discussed in this article. Long-term latency of CD4 + T cells poses a significant challenge in the eradication of AIDS. AZD5582 has been found to effectively promote the expression of HIV and simian immunodeficiency virus (SIV) through the activation of atypical NF-κB signaling in mouse and rhesus monkey models, a finding that provides a basis for combining AZD5582 with HIV-removing drugs to eradicate AIDS.^613^

Promoting ongoing research from the laboratory to the clinic is a shared objective among scientists. In the 40 years of research on NF-κB signaling, an increasing number of promising targets have been identified, leading to the development of corresponding therapeutics that have either entered clinical trials or been approved for disease treatment. It is challenging to tackle the issue of how to promote the rapid and consistent application of cellular and animal studies to human applications. The diversity of the disease spectrum and inter-species variability are significant obstacles. More advanced and closer to the real human environment research methodologies are required, encompassing spatial multi-omics, single-cell sequencing, organoids, genetically engineered animal models, 3D bioprinting, and other cutting-edge techniques. A recent study utilizing time-dependent multi-omics and single-cell RNA sequencing revealed heterogeneity in Rel and RelA-mediated gene expression and specific responses, and has demonstrated that their functional antagonism arises from co-expression in the nucleus.^614^

While we cannot overlook the potent potential of NF-κB signaling, we also need to confront the latent apprehensions underlying it. As described in this article, therapies targeting NF-κB signaling will inevitably elicit side effects, given its involvement in a diverse array of biological processes. Therefore, precise drug design, synthesis, and delivery procedures are imperative, and the utilization of nanomaterials will be pivotal in facilitating this process. Future research needs to think about how to improve the efficiency of targeting NF-κB signaling therapy while mitigating potential adverse effects, thereby achieving a significant breakthrough in the field of immunotherapy.
